# Neurotensin analogs by fluoroglycosylation at *N*^ω^-carbamoylated arginines for PET imaging of NTS1-positive tumors

**DOI:** 10.1038/s41598-022-19296-0

**Published:** 2022-09-02

**Authors:** Lisa Schindler, Katrin Wohlfahrt, Lara Gluhacevic von Krüchten, Olaf Prante, Max Keller, Simone Maschauer

**Affiliations:** 1 Faculty of Chemistry and Pharmacy, Institute of Pharmacy, University of Regensburg, Universitätsstrasse 31, 93053 Regensburg, Germany; 2 Department of Nuclear Medicine, Molecular Imaging and Radiochemistry, Friedrich-Alexander-Universität Erlangen-Nürnberg (FAU), Schwabachanlage 12, 91054 Erlangen, Germany; 3 Present Address: Hennig Arzneimittel GmbH & Co KG, Liebigstr. 1-2, 65439 Flörsheim am Main, Germany

**Keywords:** Cancer imaging, Preclinical research, Drug development

## Abstract

Since neurotensin (NT) receptors of subtype-1 (NTS1) are expressed by different types of malignant tumors, such as pancreatic adenocarcinoma, colorectal and prostate carcinoma, they represent an interesting target for tumor imaging by positron emission tomography (PET) and endoradiotherapy. Previously reported neurotensin-derived NTS1 ligands for PET were radiolabeled by modification and prelongation of the N-terminus of NT(8–13) peptide analogs. In this study, we demonstrate that modifying Arg^8^ or Arg^9^ by *N*^ω^-carbamoylation and subsequent fluoroglycosylation provides a suitable approach for the development of NT(8–13) analogs as PET imaging agents. The *N*^ω^-carbamoylated and fluoroglycosylated NT(8–13) analogs retained high NTS1 affinity in the one-digit nanomolar range as well as high metabolic stability in vitro. In vivo, the radioligand **[**^**18**^**F]21** demonstrated favorable biokinetics in HT-29 tumor-bearing mice with high tumor uptake and high retention, predominantly renal clearance, and fast wash-out from blood and other non-target tissues. Therefore, **[**^**18**^**F]21** has the potential to be used as molecular probe for the imaging of NTS1-expressing tumors by PET.

## Introduction

**Figure 1:**
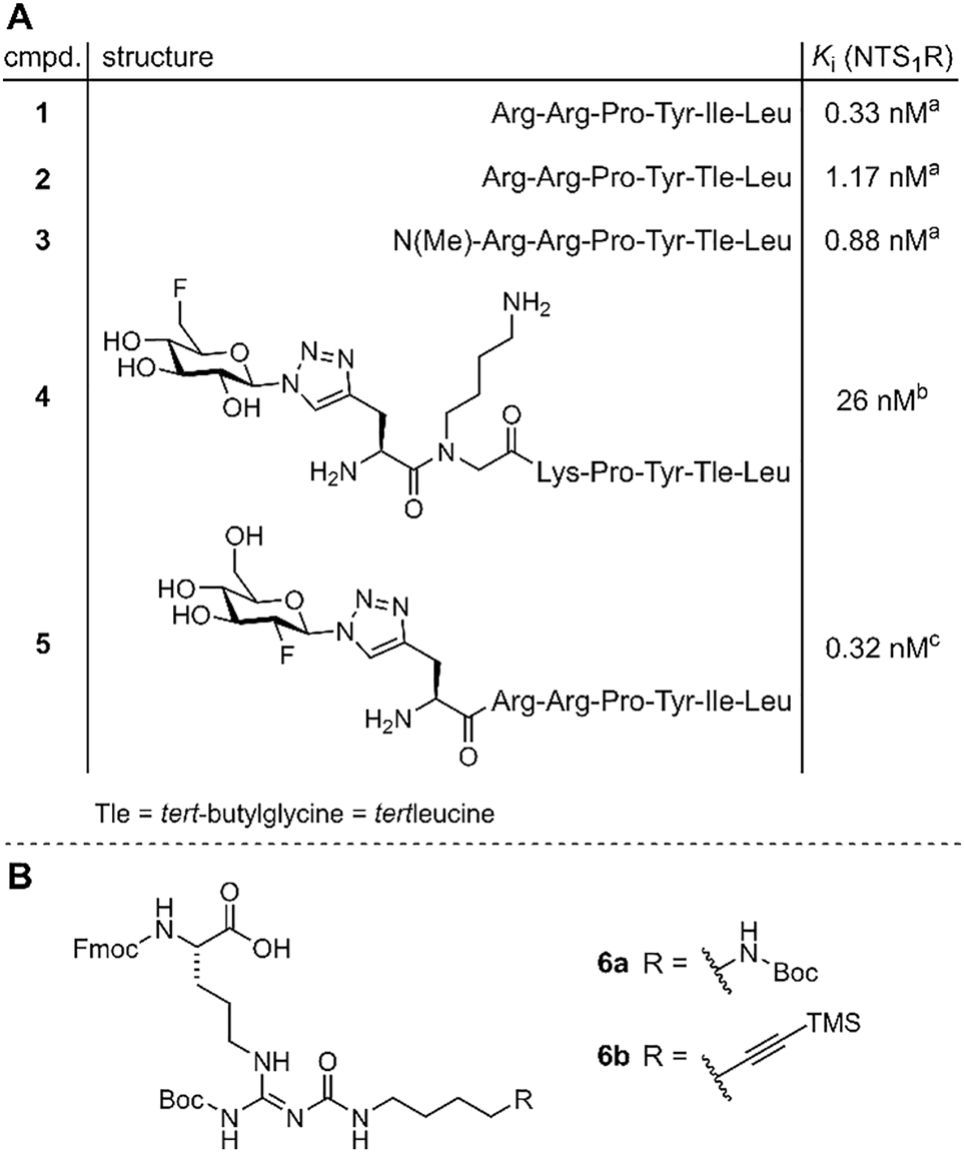
(**A**) Structures and NTS1 affinities of NT(8–13) (**1**) and reported NT(8–13)-derivatives **2**–**5**. (**B**) Structures of the previously reported arginine building blocks **6a**^23^ and **6b**^24^ which were used in this work for the synthesis of (potential) PET ligands derived from **1**. ^a^Schindler et al.^13^; ^b^Maschauer et al.^14^; ^c^Maschauer et al.^15^.

The neurotensin receptor 1 (NTS1), belonging to the class A of G-protein coupled receptors, was reported to be expressed by various types of malignant tumors, including pancreatic adenocarcinoma, colorectal and prostate carcinoma^1–6^. Therefore, the NTS1 represents an interesting target for tumor imaging by positron emission tomography (PET) and endoradiotherapy. The primary endogenous agonist of the NTS1 is the tridecapeptide neurotensin, acting as a local hormone in the gastrointestinal tract and as a neurotransmitter and neuromodulator in the central nervous system^7–9^. As the C-terminal hexapeptide sequence of neurotensin (NT(8–13) (**1**), Fig. 1A) exhibits biological activity comparable to that of neurotensin^10^, this hexapeptide has served as a lead structure for the development of a large variety of radioligand candidates for PET^11^. For in vivo applications, it is well-known that the peptide backbone of **1** needs to be stabilized against proteolytic degradation, occurring at the N-terminal and C-terminal site. Following the strategy of N-methylation of Arg^8^ and C-terminal stabilization by the introduction of Tle^12^ instead of Ile^12^^12^, we recently studied the stability of the NT(8–13) derivatives **2** and **3** (Fig. 1A), conforming that **2** was readily degraded in human plasma by enzymatic cleavage of the Arg^8^-Arg^9^ bond, whereby **3** exhibited high stability^13^. In the case of the previously described NTS1 PET ligand **4** (Fig. 1A)^14^, the N-terminal part of the peptide was stabilized by replacement of Arg^8^-Arg^9^ with NLys^8^-Lys^9^ and prolongation at the N-terminus by a fluoroglucosyl-triazolylmethyl glycine derivative. However, these structural modifications resulted in a considerable reduction in NTS1 affinity when compared to **2** or **3** (Fig. 1A). The reported NT(8–13)-derived potential PET ligand **5** is, like **4**, N-terminally fluoroglucosylated, but contains Arg^8^-Arg^9^ instead of NLys^8^-Lys^9^ and Ile instead of Tle. Compound **5** exhibits high NTS1 affinity, however, it represents no useful PET ligand candidate due to low in vitro stability in human serum^15^.

The vast majority of previously reported neurotensin-derived NTS1 PET ligands (including **4**, Fig. 1A) have in common that the prosthetic group or chelator for radiolabeling with or without a linker was attached to the N-terminus of the respective NT(8–13) peptide analogs, and in most cases, at least one arginine (Arg^8^ or Arg^9^) was replaced by lysine^14–17^.

Based on our previous work on *N*^ω^-carbamoylation of Arg^8^ or Arg^9^ for the design of fluorescence-labeled NT(8–13) analogs^18^, we herein present a series of NT(8–13) analogs containing *N*^ω^-carbamoylated Arg^8^ or Arg^9^, respectively, to allow for fluoroglycosylation in these positions. We previously demonstrated that ^18^F-fluoroglycosylation of peptides positively influences their in vivo clearance behaviour^15,19,20^, and it has been frequently shown that glycosylation is an effective approach to improve the in vivo blood stability and membrane permeability of peptides^21,22^. With the aim of combining *N*^ω^-carbamoylation with subsequent chemoselective fluoroglycosylation, the previously described arginine building blocks **6a**^23^ or **6b**^24^ (Fig. 1B) were incorporated into the NT(8–13) analogs **2** and **3** instead of Arg^8^ or Arg^9^. The resulting series of NT(8–13) analogs, containing the fluoroglycosylated peptides **11**–**13**, **20** and **21** as NTS1 PET ligand candidates, were studied in vitro regarding their NTS1 affinity as well as stability in blood plasma. Finally, the ^18^F-labeled glycopeptide **[**^**18**^**F]21** was prepared by click chemistry-based ^18^F-fluoroglycosylation and evaluated in a tumor mouse model by small animal PET imaging studies.

## Results

### Chemistry

**Figure 2:**
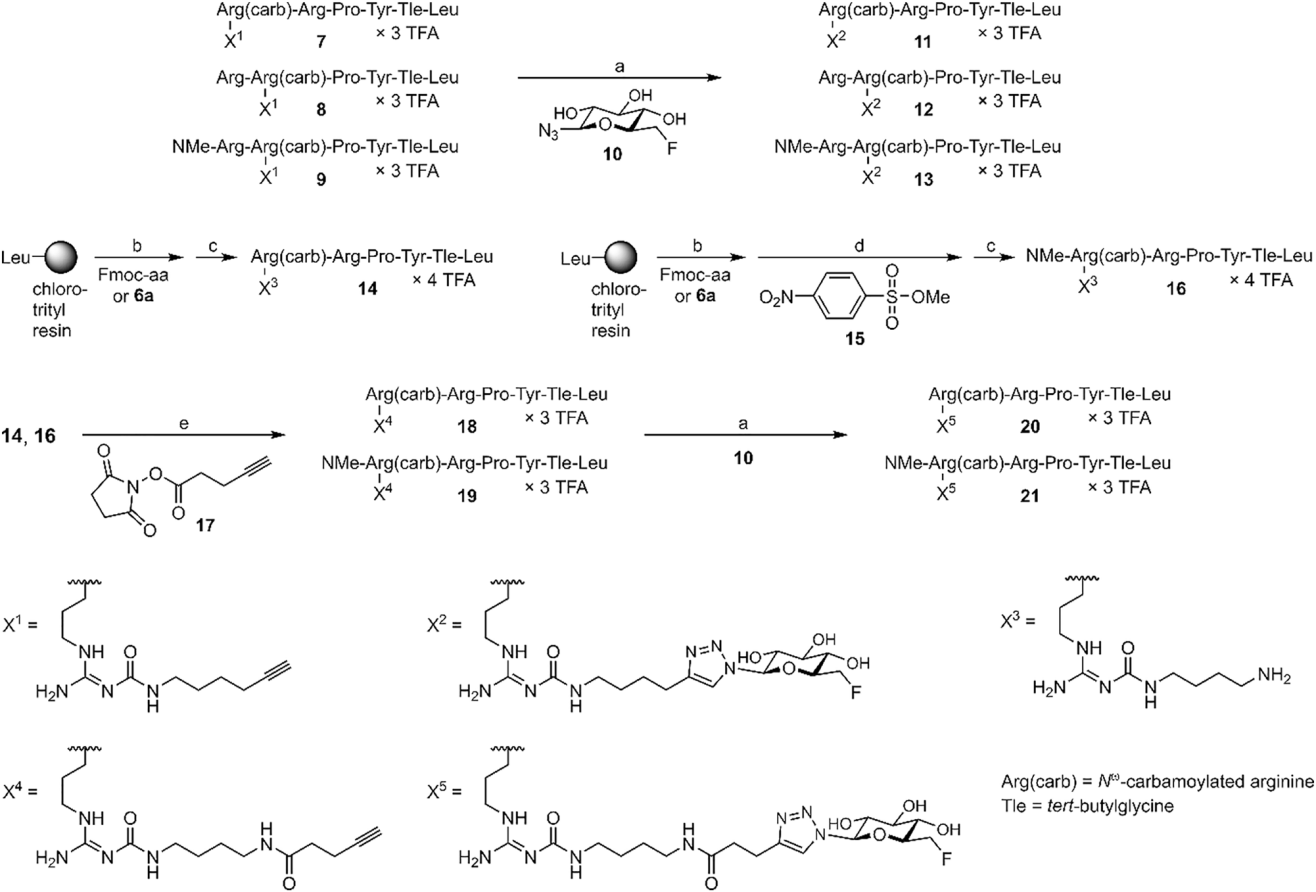
Synthesis of the glycosylated and fluorinated potential NTS1 PET ligands **11**–**13**, **20** and **21**, which were obtained by CuAAC reaction of alkyne-functionalized NT(8–13) derivatives (**7**, **8**, **9**, **18**, **19**) to 6-deoxy-6-fluoro-β-D-glucosyl azide **10**. Reagents and conditions: (**a**) CuSO_4_, sodium ascorbate, solvent: PBS/NMP (1:1 v/v) or EtOH/PBS (1:9 v/v), rt, 30 min, 45% (**11**), 45% (**12**), 68% (**13**), 25% (**20**), 79% (**21**); (**b**) Fmoc strategy SPPS using HBTU/HOBt and DIPEA, solvent: DMF/NMP (80:20 v/v), 35 °C, 2 × 1 h or 2 × 2 h, Fmoc-deprotection: 20% piperidine in DMF/NMP (80:20 v/v), rt, 2 × 8–10 min; (**c**) (1) hexafluoro-2-propanol (HFIP)/CH_2_Cl_2_ (1:3 v/v), rt, 2 × 20 min, (2) TFA/H_2_O (95:5 v/v), rt, 3 h; (**d**) (1) collidine, 2-nitrobenzenesulfonylchloride, solvent: CH_2_Cl_2_, rt, 2 h, (2) MTBD, solvent: DMF, rt, 30 min, (3) DBU, 2-mercaptoethanol, solvent: DMF, rt, 30 min; (**e**) DIPEA, solvent: DMF/NMP (80:20 v/v), rt, 30–55 min, 59% (**18**), 90% (**19**); overall yields of **14** and **16**: 49% and 45%, respectively.

The synthesis of the potential NTS1 PET ligands **11**–**13**, **20** and **21** is outlined in Fig. 2. The alkyne-functionalized precursor peptides **7**–**9**, containing a modified arginine derived from building block **6b**^24^ (Fig. 1B) in position 8 (**7**) or 9 (**8**, **9**), were obtained by the strategy of solid-phase peptide synthesis (SPPS) using Fmoc protecting groups, following a previously described procedure^23^. For the incorporation of the *N*^α^-methylated arginine in peptide **9**, commercially available Fmoc-N-Me-Arg(Pbf)-OH was used. Conjugation of **7**–**9** to 6-deoxy-6-fluoro-β-D-glucosyl azide (**10**)^19^ by copper(I)-catalyzed azide-alkyne cycloaddition (CuAAC) yielded the potential NTS1 PET ligands **11**–**13** (Fig. 2). The amino-functionalized precursor peptides **14** and **16**, both containing a modified arginine derived from **6a**^23^ (Fig. 1B) in position 8, but differing with respect to N-terminal methylation (non-methylated: **14**, N-terminally methylated: **16**), were also prepared by Fmoc SPPS. The N-terminal methyl group in peptide **16** was introduced after the coupling and Fmoc deprotection of arginine building block **6a** while the peptide was still attached to the solid support, applying a procedure reported by Miller et al.^25^ For this purpose, the resin-bound peptide was treated with collidine and 2-nitrobenzenesulfonylchloride followed by treatment with the methylating reagent 4-nitrobenzenesulfonic acid methylester (**15**) and the base MTBD. Deprotection of the secondary amine using DBU and 2-mercaptoethanol, and subsequent cleavage from the resin and side chain deprotection yielded **16** in an overall yield of 45% (Fig. 2).

The amino-functionalized precursor peptides **14** and **16** were transformed to the alkyne-functionalized peptides **18** and **19**, respectively, by treatment with the succinimidyl ester of pentyn-4-oic acid (**17**^26^). Conjugation of **18** and **19** with glycosyl azide **10** by CuAAC afforded the potential NTS1 PET ligands **20** and **21**. Purification by preparative reversed-phase HPLC yielded the series of PET ligand candidates (**11**–**13**, **20**, **21**) with a purity of ≥ 98% (detection at 220 nm). It should be noted that in all synthesized NT(8–13) derivatives, *tert*-butylglycine (*tert-*leucine, Tle) was incorporated instead of Ile^12^, as this modification results in a considerable stabilization of the C-terminus against proteolytic degradation^12,13^.

### In vitro characterization

NTS1 affinities of the precursor peptides **7**–**9**, **14**, **16**, **18** and **19** as well as of the potential PET ligands **11**–**13**, **20** and **21** were determined by competitive receptor binding experiments on human HT-29 colon carcinoma cells stably expressing NTS1, using the previously reported tritium-labeled NT(8–13) derivative [^3^H]UR-MK300^23^ as radioligand. All peptides, precursors and potential PET ligands, displayed *K*_i_ values in the single-digit nanomolar range (Table 1), demonstrating that *N*^ω^-carbamoylation at Arg^8^ or Arg^9^ and conjugation to the sugar moiety reduced the affinity for NTS1 only slightly, by a factor of 3–7, when compared to the NTS1 affinities of **2** and **3**.

**Table 1:** NTS1 affinities of **1**–**3**, **7**–**9**, **11**–**14**, **16** and **18**–**21** were determined by radioligand competition binding with [^3^H]UR-MK300 at intact HT-29 cells (*K*_d_ = 0.55 nM^13^, c = 1 nM; see Supplementary Figs. S1 and S2).

Compound	p*K*_i_ ± SEM / *K*_i_ [nM]
**1**	9.49 ± 0.03 / 0.33^13^
**2**	8.93 ± 0.0002 / 1.2^13^
**3**	9.07 ± 0.06 / 0.88^13^
**7**	8.36 ± 0.12 / 4.6
**8**	8.03 ± 0.03 / 9.4
**9**	8.13 ± 0.13 / 7.7
**11**	8.34 ± 0.04 / 4.6
**12**	8.26 ± 0.10 / 5.8
**13**	8.21 ± 0.04 / 6.2
**14**	8.69 ± 0.09 / 2.2
**16**	8.55 ± 0.03 / 2.8
**18**	8.37 ± 0.11 / 4.4
**19**	8.12 ± 0.04 / 7.7
**20**	8.60 ± 0.01 / 2.5
**21**	8.39 ± 0.08 / 4.3

Data are given as mean values ± SEM (p*K*_i_) or mean values (*K*_i_) from two (**1**, **2**, **7**, **9**, **11**, **13**, **18**, **20**), three (**8**, **12**, **14**, **16**), four (**3**, **19**) or five (**21**) independent experiments, each performed in triplicate.

The stabilities in human plasma of the non-methylated peptides **11**, **12**, **14**, **18** and **20** were considerably lower compared to the methylated peptides **13**, **16**, **19** and **21** (Table 2), showing that the proteolytic stability was significantly increased by the introduction of a methyl group at the amino-terminus of the peptides, as expected. The potential PET ligand **21**, being most favorable with respect to in vitro stability in human plasma and adequate NTS1 affinity (Tables 1 and 2) was chosen as a promising candidate for ^18^F-labeling and in vivo tumor imaging studies. In this context, it is worth mentioning that the favoured peptide **21** also showed excellent in vitro stability in mouse plasma (Table 2).

**Table 2:** In vitro plasma stabilities of **11–14, 16** and **18–21** determined at 37 °C.

Compd	% intact peptide in human plasma after the given incubation time						
	10 min	25 min	1 h	2 h	6 h	24 h	48 h
**11**	72 ± 1	24 ± 1	1.1 ± 0.1	< 1	n.d	n.d	n.d
**12**	86 ± 1	28 ± 1	< 1	< 1	n.d	n.d	n.d
**13**	n.d	n.d	n.d	> 99	> 99	> 99	96 ± 1
**14**	50 ± 2	n.d	< 1	n.d	< 1	< 1	n.d
**16**	n.d	n.d	> 99	n.d	> 99	> 99	> 99
**18**	59 ± 1	n.d	< 1	n.d	< 1	< 1	n.d
**19**	n.d	n.d	> 99	n.d	> 99	> 99	99 ± 1
**20**	78 ± 1	n.d	6.4 ± 0.1	n.d	n.d	< 1	n.d
**21**	n.d	n.d	> 99	n.d	> 99	> 99	99 ± 1

Compd	% intact peptide in mouse plasma after the given incubation time						
	10 min	25 min	1 h	2 h	6 h	24 h	48 h
**21**	> 99	n.d	> 99	> 99	> 99	> 99	> 99

The initial concentration of each peptide in human or mouse plasma/PBS (1:2 v/v) was 100 µM. Data represent mean values (± SEM) from three independent experiments (SEM not given when no decomposition was observed).

### Radiosynthesis

**Figure 3:**
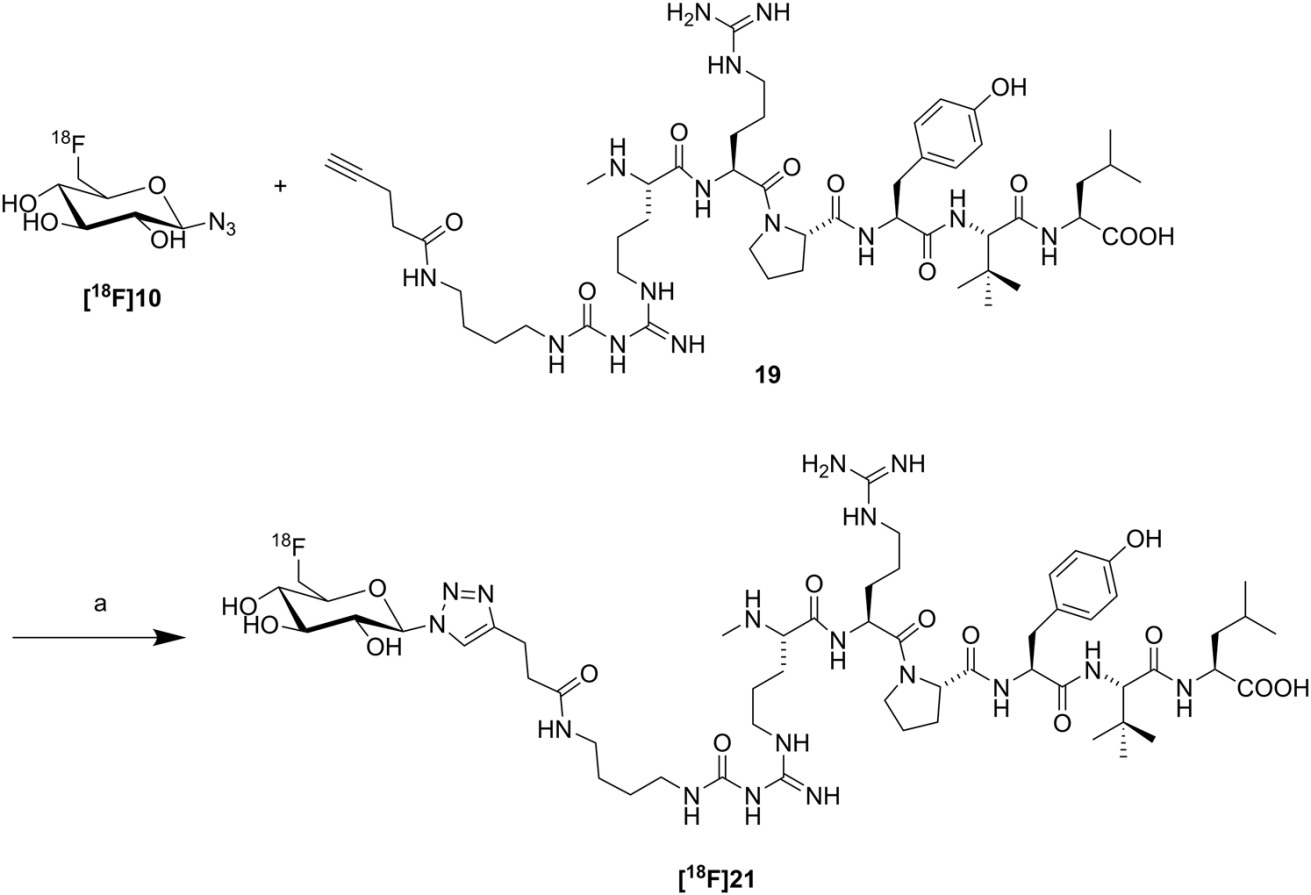
Radiosynthesis of **[**^**18**^**F]21.** Reagents and conditions: (**a**) Cu(OAc)_2_, THPTA, sodium ascorbate, phosphate buffer pH 8, 60 °C, 10 min, 92% RCY.

The nucleophilic ^18^F-for-tosylate substitution on triacetylated 6-*O*-tosyl-glucosyl azide with subsequent deacetylation to achieve 6-deoxy-6-[^18^F]fluoroglucosyl azide **[**^**18**^**F]10**^19^ (Fig. 3) has been proven to be a reliable and robust ^18^F-synthesis in our laboratory, provided that utmost caution is given to the purity of the tosylate precursor^14,20,27^. The two-step radiosynthesis of **[**^**18**^**F]21** required the ^18^F-synthesis of glycosyl azide **[**^**18**^**F]10**, which was obtained in an activity yield (AY) of 40–45% after a total synthesis time of about 30 min, and subsequent use of alkyne **19** for CuAAC with **[**^**18**^**F]10** in the presence of Cu(OAc)_2_, tris(3-hydroxypropyltriazolyl)methylamine (THPTA) and sodium ascorbate in phosphate buffer (pH 8) (Fig. 3). The radiochemical yield (RCY) of **[**^**18**^**F]21** was excellent (92% after 10 min). After isolation by semipreparative HPLC (see Supplementary Fig. S4), the total radiosynthesis starting from [^18^F]fluoride gave **[**^**18**^**F]21** in high radiochemical purity of > 99%, molar activities of 75–130 GBq/μmol (n = 5), and an AY of 20–23% in a synthesis time of 65–70 min.

### In vitro characterization and in vivo stability in blood of [^18^F]21

The logD_7.4_ of **[**^**18**^**F]21** was determined to be − 3.1, therefore showing high hydrophilicity, as expected. Accordingly, the binding of **[**^**18**^**F]21** to plasma proteins in vitro was low with only 10% of the protein-bound fraction (Table 3). As determined for reference compound **21** (Table 2), the stability of the radiotracer **[**^**18**^**F]21** in human serum and plasma in vitro was confirmed to be high as well (Table 3). After 160 min, the HPLC analysis showed degradation products of only 2% in serum and 3% in plasma (Supplementary Fig. S5). In addition, the HPLC analysis of a blood sample from one mouse, taken at 10 min p.i. of **[**^**18**^**F]21**, revealed 30% of intact tracer in the blood (Table 3). At 20 min p.i., no intact radiotracer was detectable in the blood anymore (Supplementary Fig. S5).

**Table 3:** Summary of in vitro properties and in vitro and in vivo stability of **[**^**18**^**F]21** (see also Supplementary Fig. S5).

logD_7.4_	Plasma protein binding	Stability in human serum and plasma (in vitro, after 60 min)	Stability in mouse blood (in vivo, 10 min p.i.)
− 3.1 ± 0.1 (n = 3)	10%	99%	30%

### In vivo characterization of [^18^F]21

**Figure 4:**
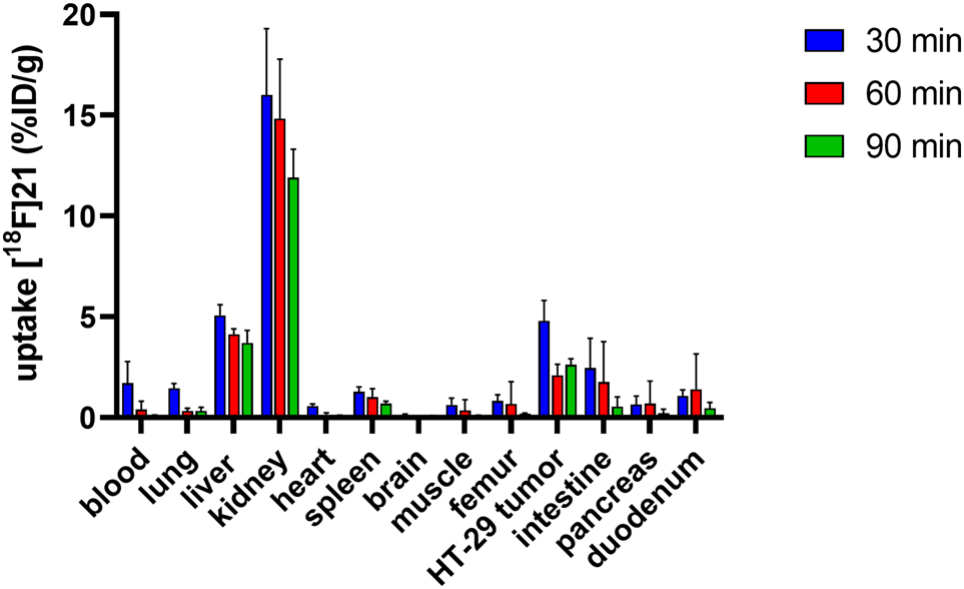
Biodistribution of **[**^**18**^**F]21** in HT-29 bearing nude mice at 30, 60 and 90 min p.i. Each bar represents the mean value ± standard deviation as determined from four independent animals per studied time point.

The biodistribution of **[**^**18**^**F]21** was studied in subcutaneous xenotransplanted HT-29 tumor-bearing mice. Mice were intravenously injected with **[**^**18**^**F]21**, dissected at 30, 60 and 90 min p.i. and organs of interest were measured for radioactivity (Fig. 4 and Supplementary Table S3). The highest uptake (12–16%ID/g) was determined in the kidneys at all time points, indicating predominant renal clearance of **[**^**18**^**F]21**. The liver showed moderate uptake values of 4–5%ID/g with a slow washout. The tumor uptake value was 5%ID/g at 30 min p.i. and about 2–3%ID/g at later time points with excellent tumor retention of **[**^**18**^**F]21** from 60 to 90 min p.i. The washout from blood was fast (2%ID/g after 30 min to 0.4%ID/g after 60 min and 0.1%ID/g after 90 min) leading to high tumor-to-blood ratios, increasing from 3 (30 min) to 30 at 90 min p.i. The tumor-to-muscle ratios were in the same range as the tumor-to-blood ratios (Supplementary Table S3).

### PET imaging

**Figure 5:**
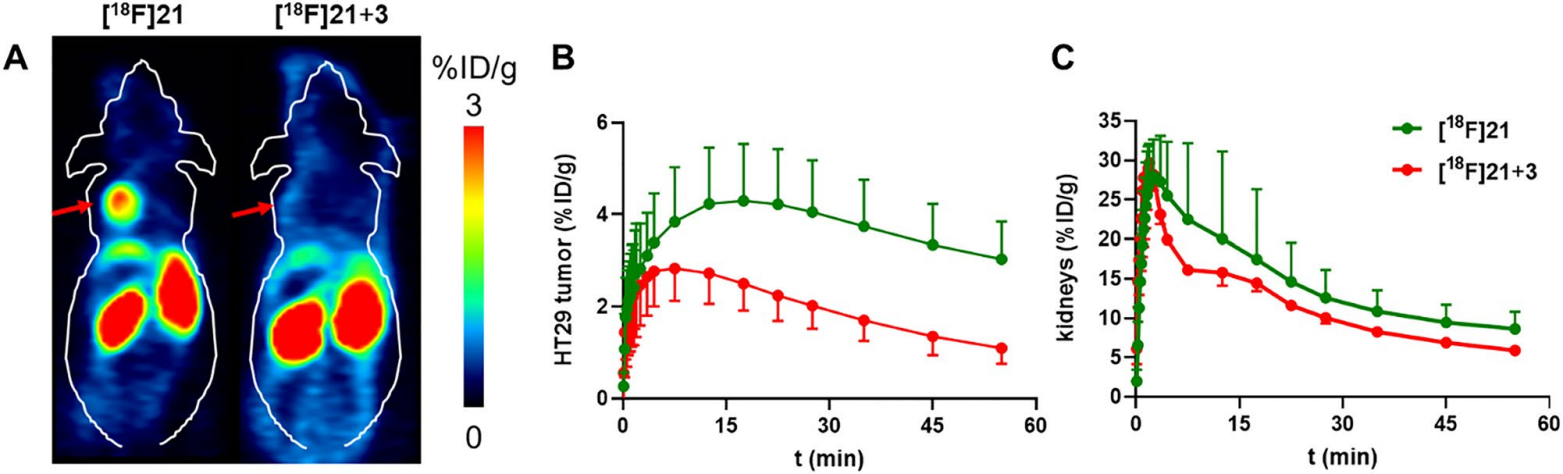
(**A**) Representative coronal PET image 50–60 min p.i. from a HT-29 tumor bearing mouse injected with **[**^**18**^**F]21** (left) and, on the following day, with **[**^**18**^**F]21** together with **3** (100 nmol, right). Red arrows indicate the tumor. B, C: Time activity curves of **[**^**18**^**F]21** in HT-29 tumors (**B**) and kidneys (**C**) in HT-29 tumor-bearing mice as determined by PET. Each point represents the mean ± standard deviation in %ID/g from animals injected with **[**^**18**^**F]21** (n = 8) and from animals coinjected with **[**^**18**^**F]21** and** 3** (100 nmol/animal, n = 4).

HT-29 tumor-bearing mice were injected with **[**^**18**^**F]21** and dynamic PET scans from 0 to 60 min were conducted to verify the specific uptake of **[**^**18**^**F]21** in NTS1-positive HT-29 tumors in vivo. Coinjections of **[**^**18**^**F]21** together with **3** as a competitive ligand were performed to define nonspecific tumor uptake of **[**^**18**^**F]21**. The highly specific uptake of **[**^**18**^**F]21** in the tumors could be demonstrated by comparing the mean tumor uptake value of animals at 60 min p.i. of **[**^**18**^**F]21 (**3.0 ± 0.8%ID/g, n = 8) to that of coninjected (**[**^**18**^**F]21** + **3**) animals (1.1 ± 0.3%ID/g, n = 4), indicating a significant 63% decrease in uptake in the tumor region (Fig. 5A). The time-activity curve for tumor uptake of **[**^**18**^**F]21** is depicted in Fig. 5B, showing the highest tumor uptake of **[**^**18**^**F]21** of 4.3 ± 1.2%ID/g at 15–20 min p.i. with a slow washout to 3.0 ± 0.8%ID/g over time, whereas the nonspecific uptake of **[**^**18**^**F]21** was 2.5 ± 0.6%ID/g at 15–20 min p.i. with washout to 1.1 ± 0.3%ID/g at 60 min p.i. (Fig. 5B). Non-target organs, such as the kidneys, showed no specific uptake (Fig. 5C).

## Discussion

Neurotensin receptors are expressed on a variety of tumor entities and therefore, have been identified as a target for diagnostic imaging as well as therapy of these tumors. Until now, a large variety of neurotensin receptor radioligands were developed, most of them based on the endogenous peptide neurotensin^11^. Among those, the vast majority of peptide tracers for PET were designed by modification and prolongation of the N-terminus of the respective peptide, to introduce a chelator, such as DOTA (1,4,7,10-tetraazacyclododecane-1,4,7,10-tetraacetic acid) or NOTA (1,4,7-triazacyclononane-1,4,7-triacetic acid) for radiolabeling with gallium-68 as positron emitter. Gallium-68 has the advantage of generally easy and fast radiolabeling in high yields and good availability of the radionuclide; however, fluorine-18 is superior regarding half-life (109 min for fluorine-18 vs. 68 min for gallium-68) and lower radiation exposure for the patient due to lower maximal decay energy also resulting in PET diagnostics with higher spatial resolution and sensitivity. A single batch of an ^18^F-labeled radiopharmaceutical can be applied to treat more patients than with a single Ga-68 production batch. Therefore, the availability of ^18^F-labeled NTS1 tracers for diagnostic imaging by PET, especially for patients with pancreatic cancer, is important.

To extend the existing portfolio of NTS1 ligands for PET, we aimed at developing ^18^F-labeled NT(8–13) analogs by *N*^ω^-carbamoylation of Arg^8^ or Arg^9^ applying building blocks **6a** or **6b**, thereby opening the possibility for regiospecific ^18^F-fluoroglycosylation at Arg^8^ or Arg^9^. It is well known that carbamoylation contributes to the molecular ageing of proteins in vivo and has important effects on the progression of chronic kidney disease^28^. For example, carbamoylation of albumin resulted in altered albumin transport in rats, leading to significantly increased vascular clearance^29^. Thus, we assumed that carbamoylation together with fluoroglycosylation could be feasible to achieve peptide radiotracers with fast clearance from blood and reduced renal uptake.

From the synthesized series of carbamoylated NTS1 peptide ligands in this work, the N-terminally methylated (Me-Arg^8^-containing) glycopeptides **13** and **21** showed high affinities towards NTS1 in the one-digit nanomolar range together with excellent in vitro stabilities over 48 h. Due to the higher NTS1 affinity of **21** compared to **13**, this glycopeptide was chosen for ^18^F-labeling and further evaluation in vitro and in vivo. The radiosynthesis of **[**^**18**^**F]21** by ^18^F-fluoroglycosylation was straightforward with high yield and provided **[**^**18**^**F]21** in high radiochemical purity. In vitro, **[**^**18**^**F]21** showed high stability in human serum and plasma with almost no degradation over 160 min. The lipophilicity was very low (logD_7.4_ = − 3.1), therefore, renal clearance in vivo could be expected, as it is common for NT analogs. The binding to plasma proteins in blood was also low (10%), which is also expected for small hydrophilic NT peptides.

To date, there are very few publications on ^18^F-labeled peptide tracers for NTS1 that demonstrate sufficient stability for in vivo experiments. Among them are **[**^**18**^**F]4**^14^ and its 2-[^18^F]fluorodeoxy congener^15^, both based on the metabolically stable sequence Pra-NLys-Lys-Pro-Tyr-Tle-Leu, as well as the Al^18^F-labeled NT derivative based on the sequence Ac-Lys(NOTA)-Pro-NMeArg-Arg-Pro-Tyr-Tle-Leu ([^18^F]AlF-NOTA-NT)^30^ (see Supplementary Fig. S3 for comparison). Due to the use of different animal models with different tumors, it appears difficult to compare the previously published ^18^F-labeled NTS1 peptides with **[**^**18**^**F]21**. However, the respective tumor-to-blood ratio of the tracers could be considered for comparison to estimate the signal-to-background ratios of PET images obtained by the respective ^18^F-labeled NTS1 peptides.

Compared to our previously published peptide **[**^**18**^**F]4**^14^ (Pra(6-[^18^F]FGlc)-NLys^8^-Lys^9^-Pro^10^-Tyr^11^-Tle^12^-Leu^13^, Fig. 1A), the tumor uptake of **[**^**18**^**F]21** was 3–fivefold higher (5%ID/g) and **[**^**18**^**F]21** revealed improved tumor retention at 60 to 90 min p.i. (2.1–2.6%ID/g). The initial renal uptake of **[**^**18**^**F]21** was significantly reduced, however, only moderate washout from kidneys was observed (16–12%ID/g from 30 to 90 min p.i. vs. 31–19%ID/g from 10 to 60 min p.i. for **[**^**18**^**F]4**^14^). The relatively low uptake in kidneys at late time points after injection is similar for both 6-deoxy-6-[^18^F]fluoroglycosyl compounds. This observation could be related to a role of the sodium-dependent glucose transporter (SGLT) in the kidney, as also described for 6-deoxy-6-fluoroglucose^31^. A large number of other published 6-deoxy-6-[^18^F]fluoroglycosylated tracers show equally low uptake in kidneys^32^, however, the role of SGLT for the clearance of 6-deoxy-6-[^18^F]fluoroglycosyl conjugates through kidneys remains to be elucidated.

In contrast to **[**^**18**^**F]4**^14^, very high tumor-to-blood and tumor-to-muscle ratios were obtained by **[**^**18**^**F]21**, increasing over time after tracer injection to reach a ratio of 30 at 90 min p.i., suggesting excellent signal-to-background ratios in PET imaging studies. It should be mentioned that, to our knowledge, a tumor-to-blood ratio of 30 has not been previously achieved with any other published ^18^F-labeled peptide ligand for NTS1. The PET scans of mice injected with **[**^**18**^**F]21** confirmed the high-contrast tumor imaging, in which the tumor is very clearly delineated from the background (Fig. 5A). The specificity of the tumor uptake of **[**^**18**^**F]21** was proven by displacement studies with coinjection of high-affinity and metabolically stable NTS1 ligand **3** together with **[**^**18**^**F]21**, demonstrating that nonspecific binding of **[**^**18**^**F]21** in the tumor is negligible.

Recently, Wang et al. reported on the synthesis of [^18^F]AlF-NOTA-NT^30^. They introduced the chelator NOTA to the formerly published “NT20.3” sequence (Ac-Lys(NOTA)-Pro-NMeArg-Arg-Pro-Tyr-Tle-Leu) and radiolabeled it with Al[^18^F]F. The resulting [^18^F]AlF-NOTA-NT showed high NTS1 affinity (IC_50_ = 2.6 nM). PET scans of AsPC-1 and Panc-1 tumor-bearing mice at 1 h p.i. demonstrated specific tumor uptake with 3–4%ID/g and tumor-to-muscle ratios of 7–8, which slightly decreased over 4 h to 5–6. As mentioned above, a direct comparison between [^18^F]AlF-NOTA-NT and **[**^**18**^**F]21** is difficult due to the use of different animal models, however, the main feature of carbamoylated peptide **[**^**18**^**F]21** is the very high tumor-to-blood ratio of 30 at 90 min p.i., whereas the radiosynthesis of [^18^F]AlF-NOTA-NT is more straightforward.

## Conclusion

Taken together, we here describe the strategy to combine *N*^ω^-carbamoylation with ^18^F-fluoroglycosylation for the development of new ^18^F-labeled NT analogs with high affinity to NTS1, sufficient metabolic stability and high and specific uptake in NTS1-positive HT-29 tumors in vivo. The PET tracer **[**^**18**^**F]21** has the potential to be used as molecular probe for PET imaging of other NTS1-expressing tumors such as pancreatic adenocarcinoma. Moreover, the present study suggests that *N*^ω^-carbamoylated arginines, such as **6a**, might be useful for the preparation of chelator-conjugated, ^68^ Ga- or ^177^Lu-labeled NT(8–13) analogs with higher NTS1 affinity compared to reported chelator-bearing NTS1 ligands.

## Methods

### General

Additional information on materials, chemicals, and additional analytical data of compounds (HPLC analyses, ^1^H- and ^13^C-NMR spectra) are provided in the Supplementary Information.

### General Procedure for SPPS

Peptides were synthesized by manual SPPS according to a reported procedure^23^ with the following modifications: The resin was allowed to swell in the solvent for 45 min before the beginning of the synthesis. Protected standard amino acids (Fmoc-Arg(Pbf)-OH, Fmoc-Pro-OH, Fmoc-Tyr(*t*Bu)-OH) were used in fivefold excess, Fmoc-N-Me-Arg(Pbf)-OH was used in 3.5-fold excess, and Fmoc-Tle-OH was used in fivefold excess (**7**, **8**), fourfold excess (**9**, **16**) or 4.4-fold excess (**14**). The arginine building block **6a** was used in threefold excess and the arginine building block **6b** was used in 2.45-fold (**7**, **8**) or threefold (**9**) excess. Amino acid coupling was performed with HBTU/HOBt/DIPEA (Fmoc-Arg(Pbf)-OH, Fmoc-Pro-OH, Fmoc-Tyr(*t*Bu)-OH: 4.9/5/10 equiv., Fmoc-N-Me-Arg(Pbf)-OH: 3.45/3.5/7 equiv., Fmoc-Tle-OH: 4.9/5/10 (**7**, **8**), 3.95/4/8 (**9**, **16**) or 4.35/4.4/8.8 (**14**) equiv., **6a**: 3/3/6 equiv., **6b**: 2.2/2.2/4.4 (**7**, **8**) or 2.95/3/6 (**9**) equiv.). For the coupling of Fmoc-N-Me-Arg(Pbf)-OH and the arginine building blocks **6a** and **6b**, anhydrous solvents (DMF, NMP) were used. Except for the arginine building blocks **6a** and **6b**, “double coupling” was performed (2 × 45 min or 60 min at 35 °C). In the case of the arginine derivatives **6a** and **6b**, “single coupling” was performed with a longer reaction time (16 h at 35 °C). Peptides were cleaved off the resin with CH_2_Cl_2_/HFIP (4:1 v/v) (**7**, **8**) or CH_2_Cl_2_/HFIP (3:1 v/v) (**9**, **14**, **16**) (rt, 2 × 20 min).

### H-(***N***^ω^-5-hexynylaminocarbonyl)Arg-Arg-Pro-Tyr-2-***tert***-butyl-Gly-Leu-OH tris(hydrotrifluoroacetate) (7)

The peptide was synthesized according to the general procedure (resin**:** 43.8 mg, 0.034 mmol). The product was purified by preparative RP-HPLC (column: Kinetex-XB C18; gradient: 0 − 35 min: A2/B 92:8–47:53, *t*_R_ = 22 min). Lyophilization of the eluate afforded peptide **7** as white fluffy solid (16.9 mg, 39%). ^1^H-NMR (600 MHz, DMSO-*d*_6_): δ 0.81–0.95 (m, 15H), 1.40–1.65 (m, 12H), 1.65–1.77 (m, 3H), 1.77–1.88 (m, 3H), 1.88–2.03 (m, 1H), 2.14–2.23 (td, 2H, *J* 6.9, 2.6 Hz), 2.64–2.73 (m, 1H), 2.77 (t, 1H, *J* 2.6 Hz), 2.82–2.93 (m, 1H), 3.02–3.16 (m, 4H), 3.20–3.29 (m, 2H), 3.52–3.66 (m, 2H), 3.78–3.94 (m, 1H), 4.16–4.25 (m, 1H), 4.28 (d, 1H, *J* 9.8 Hz), 4.32–4.40 (m, 1H), 4.40–4.59 (m, 2H), 6.58–6.62 (m, 2H), 6.62–7.18 (br s, 2H, interfering with the next listed signal), 6.97–7.01 (m, 2H), 7.18–7.49 (br s, 2H), 7.49–7.57 (br s, 1H), 7.61 (d, 1H, *J* 9.3 Hz), 7.65–7.72 (m, 1H), 7.97 (d, 1H, *J* 8.1 Hz), 8.03–8.30 (m, 4H), 8.30–8.56 (m, 2H), 8.67 (d, 1H, *J* 7.5 Hz), 8.96–9.18 (br s, 1H), 9.20 (s, 1H), 10.07–10.51 (br s, 1H), 12.23–12.75 (br s, 1 H). ^13^C-NMR (150 MHz, DMSO-*d*_*6*_): δ 17.3, 21.2, 22.8, 23.5, 24.2, 24.3, 24.5, 25.2, 26.5 (3 carbon atoms), 28.1, 28.2, 28.3, 29.1, 34.8, 36.3, 38.6, 39.7, 40.1, 40.5, 46.8, 50.1, 50.4, 51.6, 54.2, 59.1, 59.2, 71.4, 84.2, 114.8 (2 carbon atoms), 116.2 (TFA), 118.2 (TFA), 127.6, 130.0 (2 carbon atoms), 153.8 (2 carbon atoms), 155.7, 156.8, 158.5 (q, *J* 31 Hz) (TFA), 168.3, 169.1, 169.8, 170.5, 171.3, 173.8. HRMS (ESI): *m/z* [*M* + 2H]^2+^ calcd. for [C_45_H_75_N_13_O_9_]^2+^ 470.7900, found 470.7912. RP‐HPLC (220 nm): > 99% (*t*_R_ = 10.3 min, *k* = 12.2). C_45_H_73_N_13_O_9_ · C_6_H_3_F_9_O_6_ (940.16 + 342.07).

### H-Arg-(***N***^ω^-5-hexynylaminocarbonyl)Arg-Pro-Tyr-2-***tert***-butyl-Gly-Leu-OH tris(hydrotrifluoroacetate) (8)

The peptide was synthesized according to the general procedure (resin: 102.2 mg, 0.081 mmol). The product was purified by preparative RP-HPLC (column: Kinetex-XB C18; gradient: 0–35 min: A2/B 92:8–47:53, *t*_R_ = 23 min). Lyophilization of the eluate afforded **8** as white fluffy solid (38.7 mg, 37%). ^1^H-NMR (600 MHz, DMSO-*d*_6_): δ 0.80–0.94 (m, 15H), 1.41–1.74 (m, 15H), 1.74–1.89 (m, 3H), 1.95–2.03 (m, 1H), 2.14–2.19 (dt, 2H, *J* 6.8, 2.7 Hz), 2.64–2.72 (m, 1H), 2.76 (t, 1H, *J* 2.2 Hz), 2.85–2.92 (m, 1H), 3.05–3.15 (m, 4H), 3.19–3.28 (m, 2H), 3.52–3.64 (m, 2H), 3.78–3.86 (m, 1H), 4.17–4.24 (m, 1H), 4.28 (d, 1H, *J* 9.5 Hz), 4.28–4.39 (m, 1H), 4.43–4.57 (m, 2H), 6.59–6.62 (m, 2H), 6.62–7.20 (br s, 2H, interfering with the next listed signal), 6.97–7.00 (m, 2H), 7.20–7.51 (br s, 2H), 7.51–7.57 (br s, 1H), 7.63 (d, 1H, *J* 9.2 Hz), 7.76 (t, 1H, *J* 6.0 Hz), 7.94 (d, 1H, *J* 7.3 Hz), 8.02–8.31 (br s, 4H), 8.33–8.57 (br s, 2H), 8.68 (d, 1H, *J* 7.9 Hz), 8.95–9.12 (br s, 1H), 9.20 (s, 1H), 10.03–10.70 (br s, 1H), 12.21–12.80 (br s, 1H). ^13^C-NMR (150 MHz, DMSO-*d*_*6*_): δ 17.3, 21.2, 22.8, 24.08, 24.14, 24.3, 25.2, 26.5 (3 carbon atoms), 28.1, 28.2, 28.4, 29.1, 34.7, 36.3, 38.6, 39.7, 40.1, 40.6, 46.8, 50.1, 50.3, 51.7, 54.2, 59.1, 59.2, 71.4, 84.2, 114.8 (2 carbon atoms), 114.8, 116.1 (TFA), 118.1 (TFA), 127.6, 130.0 (2 carbon atoms), 153.8 (2 carbon atoms), 155.7, 156.8, 158.7 (q, *J* 31 Hz) (TFA), 168.3, 169.1, 169.8, 170.5, 171.2, 173.8. HRMS (ESI): *m/z* [*M* + H]^+^ calcd. for [C_45_H_74_N_13_O_9_]^+^ 940.5727, found 940.5724. RP‐HPLC (220 nm): > 99% (*t*_R_ = 10.4 min, *k* = 12.7). C_45_H_73_N_13_O_9_ · C_6_H_3_F_9_O_6_ (940.16 + 342.07).

### ***N***^α^-(***N***^α^-methylarginyl)-***N***^ω^-(5-hexynylaminocarbonyl)Arg-Pro-Tyr-2-***tert***-butyl-Gly-Leu-OH tris(hydrotrifluoroacetate) (9)

The peptide was synthesized according to the general procedure (resin: 76 mg, 0.060 mmol). The product was purified by preparative RP-HPLC (column: Kinetex-XB C18; gradient: 0–35 min: A2/B 92:8–47:53, *t*_R_ = 24 min). Lyophilization of the eluate afforded **9** as white fluffy solid (44 mg, 57%). ^1^H-NMR (600 MHz, DMSO-*d*_6_): δ 0.80–0.94 (m, 15H), 1.41–1.77 (m, 15H), 1.77–1.88 (m, 3H), 1.95–2.04 (m, 1H), 2.13–2.21 (m, 2H), 2.47 (s, 3H), 2.64–2.72 (m, 1H), 2.76 (t, 1H, *J* 2.7 Hz), 2.83–2.92 (m, 1H), 3.05–3.16 (m, 4H), 3.19–3.29 (m, 2H), 3.52–3.65 (m, 2H), 3.71–3.82 (m, 1H), 4.18–4.25 (m, 1H), 4.26–4.31 (m, 1H), 4.33–4.40 (m, 1H), 4.43–4.51 (m, 1H), 4.51–4.61 (m, 1H), 6.58–6.63 (m, 2H), 6.85–7.24 (br s, 2H, interfering with the next listed signal), 6.96–7.01 (m, 2H), 7.24–7.49 (br s, 2H), 7.53 (s, 1H), 7.63 (d, 1H, *J* 9.5 Hz), 7.82 (t, 1H, *J* 5.8 Hz), 7.94 (d, 1H, *J* 7.3 Hz), 8.19 (d, 1H, *J* 7.4 Hz), 8.37–8.65 (br s, 2H), 8.66–9.18 (m, 4H), 9.21 (s, 1H), 10.13–10.63 (br s, 1H), 12.09–12.80 (br s, 1H). ^13^C-NMR (150 MHz, DMSO-*d*_*6*_): δ 17.7, 21.6, 23.1, 24.1, 24.3, 24.6, 24.7, 25.5, 26.8 (3 carbon atoms), 27.2, 28.1, 28.4, 29.4, 31.4, 35.0, 36.5, 38.9, 39.7, 40.0, 40.3, 40.8, 47.3, 50.5, 50.9, 54.5, 59.6, 60.1, 71.6, 84.7, 115.1 (2 carbon atoms), 116.4 (TFA), 118.3 (TFA), 127.9, 130.4 (2 carbon atoms), 153.8, 153.9, 155.9, 156.8, 159.2 (q, *J* 32 Hz) (TFA), 167.3, 169.5, 170.2, 170.9, 171.7, 174.1. HRMS (ESI): *m/z* [*M* + H]^+^ calcd. for [C_46_H_76_N_13_O_9_]^+^ 954.5883, found 954.5884. RP‐HPLC (220 nm): > 99% (*t*_R_ = 11.0 min, *k* = 11.2). C_46_H_75_N_13_O_9_ · C_6_H_3_F_9_O_6_ (954.19 + 342.07).

### H-{*N*^ω^-[*N*-(4-{1-[6-deoxy-6-fluoro-β-D-glucopyranosyl]-1*H*-1,2,3-triazol-4-yl}butyl)aminocarbonyl]}Arg-Arg-Pro-Tyr-2-*tert*-butyl-Gly-Leu-OH tris(hydrotrifluoroacetate) (11)

To a solution of **7** (3.94 mg, 2.96 μmol) and 6-deoxy-6-fluoro-*β*-D-glucosyl azide (**10**) (2.45 mg, 11.8 μmol) in EtOH/PBS (1:9 v/v) (0.5 mL) were added a 0.2 M solution of copper(II)sulfate pentahydrate (17.8 μL, 3.55 μmol) in EtOH/PBS (1:9 v/v) and a 0.6 M solution of sodium ascorbate (17.8 μL, 10.7 μmol) in EtOH/PBS (1:9 v/v). The mixture was stirred at rt for 30 min (complete consumption of **7** was verified by analytical HPLC). The product was purified by preparative RP-HPLC (column: Kinetex Biphenyl; gradient: 0–30 min: A1/B 93:7–76:24, *t*_R_ = 21 min). Lyophilization of the eluate afforded **11** as white fluffy solid (1.53 mg, 45%). ^1^H-NMR (600 MHz, DMSO-*d*_6_): δ 0.79–0.96 (m, 15H), 1.46–1.65 (m, 12H), 1.65–1.76 (m, 3H), 1.76–1.91 (m, 3H), 1.94–2.07 (m, 1H), 2.63–2.71 (m, 2H), 2.84–2.92 (m, 1H), 2.82–2.93 (m, 1H), 3.05–3.16 (m, 4H), 3.23–3.28 (m, 3H), 3.40–3.47 (m, 2H), 3.50–3.66 (m, 2H), 3.67–3.78 (m, 2H), 3.78–3.86 (m, 1H), 4.17–4.25 (m, 1H), 4.28 (d, 1H, *J* 9.8 Hz), 4.31–4.39 (m, 1H), 4.42–4.66 (m, 4H), 5.35–5.51 (m, 3H), 5.57 (d, 1H, *J* 9.0 Hz), 6.56–6.63 (m, 2H), 6.63–7.08 (br s, 2H, interfering with the next listed signal), 6.97–7.01 (m, 2H), 7.08–7.48 (br s, 2H), 7.52 (s, 1H), 7.54–7.59 (m, 1H), 7.59–7.64 (d, 1H, *J* 9.4 Hz), 7.98 (d, 1H, *J* 8.1 Hz), 8.03 (s, 1H), 8–05-8.19 (br s, 3H), 8.22 (d, 1H, *J* 7.5 Hz), 8.26–8.56 (br s, 2H), 8.66 (d, 1H, *J* 7.5 Hz), 8.91–9.13 (br s, 1H), 9.18 (s, 1H), 9.79–10.05 (br s, 1H), 12.14–12.71 (br s, 1H). HRMS (ESI): *m/z* [*M* + H]^+^ calcd. for [C_51_H_84_FN_16_O_13_]^+^ 1147.6382, found 1147.6375. RP‐HPLC (220 nm): > 99% (*t*_R_ = 8.0 min, *k* = 9.5). C_51_H_83_FN_16_O_13_ · C_6_H_3_F_9_O_6_ (1147.32 + 342.07).

### H-Arg-{*N*^ω^-[*N*-(4-{1-[6-deoxy-6-fluoro-β-D-glucopyranosyl]-1*H*-1,2,3-triazol-4-yl}butyl)aminocarbonyl]}Arg-Pro-Tyr-2-*tert*-butyl-Gly-Leu-OH tris(hydrotrifluoroacetate) (12)

Compound **12** was prepared from **8** (9.7 mg, 7.57 μmol) and **10** (6.27 mg, 30.3 μmol) using the procedure for the preparation of **11**. The product was purified by preparative RP-HPLC (column: Kinetex Biphenyl; gradient: 0–35 min: A1/B 90:10–62:38, *t*_R_ = 21 min). Lyophilization of the eluate afforded **12** as white fluffy solid (8.72 mg, 77%). ^1^H-NMR (600 MHz, DMSO-*d*_6_): δ 0.78–0.96 (m, 15H), 1.44–1.76 (m, 15H), 1.76–1.91 (m, 3H), 1.94–2.06 (m, 1H), 2.63–2.68 (m, 2H), 2.84–2.93 (m, 1H), 3.07–3.16 (m, 4H), 3.23–3.29 (m, 3H), 3.43–3.45 (m, 2H), 3.56–3.61 (m, 2H), 3.69–3.82 (m, 3H), 4.17–4.26 (m, 1H), 4.28 (d, 1H, *J* 9.8 Hz), 4.31–4.38 (m, 1H), 4.44–4.66 (m, 4H), 5.29–5.54 (m, 3H), 5.56 (d, 1H, *J* 9.0 Hz), 6.59–6.63 (m, 2H), 6.63–7.10 (br s, 2H, interfering with the next listed signal), 6.97–7.01 (m, 2H), 7.10–7.51 (br s, 2H), 7.51–7.61 (m, 2 H), 7.63 (d, 1H, *J* 9.4 Hz), 7.95 (d, 1H, *J* 8.1 Hz), 8.03 (s, 1H), 8.06–8.19 (br s, 3H), 8.21 (d, 1H, *J* 7.5 Hz), 8.29–8.62 (br s, 2H), 8.67 (d, 1H, *J* 7.5 Hz), 8.85–9.09 (br s, 1H), 9.17 (s, 1H), 9.73–9.97 (br s, 1H), 12.34–12.65 (br s, 1H). HRMS (ESI): *m/z* [*M* + H]^+^ calcd. for [C_51_H_84_FN_16_O_13_]^+^ 1147.6382, found 1147.6380. RP‐HPLC (220 nm): > 99% (*t*_R_ = 8.0 min, *k* = 9.5). C_51_H_83_FN_16_O_13_ · C_6_H_3_F_9_O_6_ (1147.32 + 342.07).

### *N*^α^-(*N*^α^-methylarginyl)-{*N*^ω^-[*N*-(4-{1-[6-deoxy-6-fluoro-β-D-glucopyranosyl]-1*H*-1,2,3-triazol-4-yl}butyl)aminocarbonyl]}Arg-Pro-Tyr-2-*tert*-butyl-Gly-Leu-OH tris(hydrotrifluoroacetate) (13)

To a solution of **9** (12.0 mg, 9.26 μmol) and 6-deoxy-6-fluoro-*β*-D-glucosyl azide (**10**) (5.4 mg, 26.0 μmol) in EtOH/PBS (1:9 v/v) (0.5 mL) were added a 0.2 M solution of copper(II)sulfate pentahydrate (56.0 μL, 11.1 μmol) in EtOH/PBS (1:9 v/v) and a 0.6 M solution of sodium ascorbate (46.0 μL, 27.8 μmol) in EtOH/PBS (1:9 v/v). The mixture was stirred at rt for 30 min (complete consumption of **9** was detected by analytical HPLC) and acidified by the addition of 10% aq. TFA (2.7 μL). The product was purified by preparative RP-HPLC (column: Kinetex-XB C18; gradient: 0–35 min: A2/B 92:8–55:45, *t*_R_ = 22 min). Lyophilization of the eluate afforded **13** as white fluffy solid (9.5 mg, 68%). ^1^H-NMR (600 MHz, DMSO-*d*_6_): δ 0.77–0.98 (m, 15H), 1.42–1.76 (m, 15H), 1.78–1.90 (m, 3H), 1.95–2.04 (m, 1H), 2.43–2.47 (m, 3H), 2.63–2.70 (m, 2H), 2.82–2.94 (m, 1H), 3.04–3.17 (m, 4H), 3.21–3.29 (m, 3H), 3.41–3.46 (m, 1H), 3.51–3.65 (m, 2H), 3.65–3.86 (m, 3H), 4.16–4.25 (m, 1H), 4.25–4.32 (m, 1H), 4.32–4.39 (m, 1H), 4.42–4.65 (m, 4H), 5.36–5.44 (m, 2H), 5.46 (d, 1H, *J* 5.5 Hz), 5.56 (d, 1H, *J* 9.0 Hz), 6.57–6.66 (m, 2H), 6.69–7.13 (br s, 2H, interfering with the next listed signal), 6.97–7.01 (m, 2H), 7.13–7.45 (br s, 2H), 7.47–7.54 (m, 1 H), 7.57–7.68 (m, 2H), 7.94 (d, 1H, *J* 7.0 Hz), 8.03 (s, 1H), 8.16–8.25 (m, 1H), 8.25–8.73 (m, 3H), 8.73–9.11 (m, 4H), 9.17 (s, 1H), 9.76–10.04 (m, 1H), 12.38–12.59 (br s, 1H). HRMS (ESI): *m/z* [*M* + H]^+^ calcd. for [C_52_H_86_N_16_O_13_]^+^ 1161.6539, found 1161.6534. RP‐HPLC (220 nm): 98% (*t*_R_ = 8.4 min, *k* = 8.7). C_52_H_85_FN_16_O_13_ · C_6_H_3_F_9_O_6_ (1161.35 + 342.07).

### *N*^ω^-[(4-aminobutyl)aminocarbonyl]Arg-Arg-Pro-Tyr-2-*tert*-butyl-Gly-Leu-OH tetrakis(hydrotrifluoroacetate) (14)

The peptide was synthesized according to the general procedure (resin: 200 mg, 0.158 mmol). Purification by preparative RP-HPLC (column: Kinetex-XB C18; gradient: 0–35 min: A2/B 92:8–57:43, *t*_R_ = 18 min) yielded **14** as white fluffy solid (108.1 mg, 49%) with a HPLC purity of 95% (220 nm). A fraction (ca. 13 mg) was purified again (gradient: 0–18 min: A2/B 92:8–75:25, 18–40 min: 75:25–38:62, *t*_R_ = 19 min) yielding **14** with a HPLC purity of 97% (220 nm). ^1^H-NMR (600 MHz, DMSO-*d*_6_): δ 0.78–0.96 (m, 15H), 1.44–1.64 (m, 12H), 1.66–1.77 (m, 3H), 1.77–1.90 (m, 3H), 1.90–2.04 (m, 1H), 2.68 (dd, 1H, *J* 8.1, 14.0 Hz), 2.79 (s, 2H), 2.85–2.96 (m, 1H), 3.01–3.18 (m, 4H), 3.26 (s, 2H), 3.51–3.64 (m, 2H), 3.84 (s, 1H), 4.14–4.25 (m, 1H), 4.28 (d, 1H, *J* 9.5 Hz), 4.31–4.40 (m, 1H), 4.40–4.58 (m, 2H), 6.56–6.67 (m, 2H), 6.96–7.00 (m, 2H), 7.00–7.26 (br s, 2H), 7.26–7.56 (br s, 2H), 7.56–7.68 (m, 2H), 7.70–7.89 (m, 4H), 7.95 (d, 1H, *J* 7.8 Hz), 8.10–8.36 (m, 4H), 8.36–8.64 (m, 2H), 8.68 (d, 1H, *J* 7.4 Hz), 9.09 (br s, 1H), 9.17–9.38 (m, 1H), 10.76 (br s, 1H), 12.51 (br s, 1H). ^13^C-NMR (150 MHz, DMSO-*d*_*6*_): δ 21.2, 22.8, 23.5, 24.1, 24.3, 24.4, 24.6, 26.0, 26.5 (3 carbon atoms), 28.2, 28.3, 29.1, 34.7, 36.3, 38.5, 38.6, 39.8, 40.1, 40.5, 46.8, 50.2, 50.4, 51.6, 54.2, 59.1, 59.2, 114.8 (2 carbon atoms), 116.1 (TFA), 118.0 (TFA), 127.6, 130.0 (2 carbon atoms), 153.9, 153.9, 155.8, 156.9, 158.8 (q, *J* 32 Hz) (TFA), 168.3, 169.2, 169.8, 170.6, 171.3, 173.8. HRMS (ESI): *m/z* [*M* + 2H]^2+^ calcd. for [C_43_H_76_N_14_O_9_]^2+^ 466.2954, found 466.2962. RP-HPLC (220 nm): 97% (*t*_R_ = 5.7 min, *k* = 6.5). C_43_H_74_N_14_O_9_・C_8_H_4_F_12_O_8_ (931.14 + 456.09).

### *N*^α^-methyl-*N*^*ω*^-[(4-aminobutyl)aminocarbonyl]Arg-Arg-Pro-Tyr-2-*tert*-butyl-Gly-Leu-OH tetrakis(hydrotrifluoroacetate) (16)

The N-terminally non-methylated precursor peptide of peptide **16** was synthesized according to the general procedure (resin: 280 mg, 0.2212 mmol). N-terminal methylation: after coupling and Fmoc-deprotection of the last amino acid (arginine building block **6a**), the resin was washed with CH_2_Cl_2_ (5 ×), a solution of 2-nitrobenzenesulfonylchloride (147 mg, 0.664 mmol) and collidine (147 µL, 1.11 mmol) in CH_2_Cl_2_ (4.5 mL) was added to the resin and the mixture was shaken at rt for 2 h. The resin was washed with DMF (5 ×), and a solution of MTBD (127 µL, 0.885 mmol) and **15** (240 mg, 1.11 mmol) in DMF (5 mL) was added. After shaking at rt for 30 min, the resin was washed with DMF (3 ×) followed by the addition of a solution of DBU (165 µL, 1.11 mmol) and 2-mercaptoethanol (154 µL, 2.21 mmol) in DMF (5 mL) and shaking at rt for 30 min. After washing with DMF (5 ×), the resin was washed with K_2_CO_3_-treated CH_2_Cl_2_ and the peptide was cleaved off the resin as described in the general procedure. Purification by preparative RP-HPLC (column: Kinetex-XB C18; gradient: 0–35 min: A2/B 86:14–67:33, *t*_R_ = 15 min) afforded **16** as white fluffy solid (139.5 mg, 45%). ^1^H-NMR (600 MHz, DMSO-*d*_6_): δ 0.76–0.96 (m, 15H), 1.41–1.88 (m, 18H), 1.91–2.05 (m, 1H), 2.44–2.49 (m, 3H), 2.63–2.72 (m, 1H), 2.75–2.83 (m, 2H), 2.85–2.93 (m, 1H), 3.04–3.16 (m, 4H), 3.22–3.30 (m, 2H), 3.53–3.62 (m, 2H), 3.78–3.83 (m, 1H), 4.19–4.24 (m, 1H), 4.28 (d, 1H, *J* 9.5 Hz), 4.33–4.41 (m, 1H), 4.43–4.58 (m, 2H), 6.56–6.64 (m, 2H), 6.78–7.23 (br s, 2H, interfering with the next listed signal), 6.96–7.00 (m, 2H), 7.23–7.55 (br s, 2H), 7.55–7.69 (m, 2H), 7.69–8.03 (m, 5H), 8.22 (d, 1H, *J* 7.6 Hz), 8.31–8.71 (m, 2H), 8.71–9.46 (m, 5H), 10.54–10.86 (m, 1H), 12.49 (br s, 1H). ^13^C-NMR (150 MHz, DMSO-*d*_*6*_): δ 21.2, 22.8, 23.4, 24.2, 24.3, 24.4, 24.6, 26.0, 26.5 (3 carbon atoms), 26.9, 28.1, 29.1, 31.1, 34.8, 36.3, 38.5, 38.6, 39.8, 40.1, 40.4, 46.8, 50.2, 50.6, 54.2, 59.1, 59.2, 59.8, 114.8 (2 carbon atoms), 116.0 (TFA), 117.9 (TFA), 127.6, 130.0 (2 carbon atoms), 153.9 (2 carbon atoms), 155.8, 156.9, 158.7 (q, *J* 32 Hz) (TFA), 166.9, 169.0, 169.8, 170.5, 171.2, 173.8. HRMS (ESI): *m/z* [*M* + 2H]^2+^ calcd. for [C_44_H_78_N_14_O_9_]^2+^ 473.3033, found 473.3038. RP-HPLC (220 nm): 98% (*t*_R_ = 5.8 min, *k* = 6.6). C_44_H_76_N_14_O_9_・C_8_H_4_F_12_O_8_ (945.18 + 456.09).

### 4-Pentynoic acid succinimidyl ester (17)^26^

4-Pentynoic acid (2 g, 20.4 mmol) and *N*-hydroxysuccinimide (2.35 g, 20.4 mmol) were suspended in anhydrous CH_2_Cl_2_ (200 mL) under argon atmosphere, the mixture was cooled in an ice bath, and DCC (4.21 g, 20.4 mmol) was added under stirring. After 1 h, the ice bath was removed and stirring was continued at rt overnight. The white solid was separated by filtration and washed with CH_2_Cl_2_. The filtrate and the washings were combined, the volatiles were removed under reduced pressure, and the residue was subjected to crystallization (CH_2_Cl_2_/diethyl ether) to afford **17** as a colorless needle-like crystalline solid (1.31 g, 33%). The mother liquor was subjected to column chromatography (n-Hex/EtOAc 3:1–1:1) to obtain the residual product **17** as white solid (1.76 g, 44%). TLC: (light petroleum/EtOAc 1:1 v/v): *R*_f_ = 0.5. ^1^H-NMR (300 MHz, CDCl_3_): δ 2.04 (t, 1H, *J* 2.7 Hz), 2.57–2.63 (m, 2H), 2.83 (s, 4H), 2.84–2.90 (m, 2H). ^13^C-NMR (100 MHz, DMSO-*d*_*6*_): δ 13.4, 25.4, 29.6, 72.2, 82.0, 167.6, 170.1. HRMS (ESI): *m/z* [*M* + H]^+^ calcd. for [C_9_H_10_NO_4_]^+^ 196.0604, found 196.0605. C_9_H_9_NO_4_ (195.17).

### H-{*N*^ω^-[*N*-(4-pent-4-ynoylaminobutyl)aminocarbonyl]}Arg-Arg-Pro-Tyr-2-*tert*-butyl-Gly-Leu-OH tris(hydrotrifluoroacetate) (18)

A solution of **17** (3.95 mg, 20.3 μmol) in anhydrous DMF was added to a stirred solution of compound **14** tetrakis(hydrotrifluoroacetate) (42.15 mg, 30.4 μmol) and DIPEA (41.4 μL, 0.243 mmol) in anhydrous DMF/NMP (75:25 v/v) (250 μL) and stirring was continued at rt for 30 min. The mixture was acidified by addition of 10% aq. TFA (240 μL) and the product was purified by preparative RP-HPLC (column: Kinetex-XB C18; gradient: 0–35 min: A2/B 92:8–55:45, *t*_R_ = 23 min). Lyophilization of the eluate afforded **18** as white fluffy solid (16.2 mg, 59%). ^1^H-NMR (600 MHz, DMSO-*d*_6_): δ 0.80–0.93 (m, 15H), 1.35–1.46 (m, 4H), 1.46–1.64 (m, 8H), 1.64–1.76 (m, 3H), 1.76–1.90 (m, 3H), 1.94–2.04 (m, 1H), 2.22–2.28 (m, 2H), 2.32–2.37 (dt, 2H, *J* 7.2, 2.8 Hz), 2.64–2.71 (m, 1H), 2.74 (t, 1H, *J* 2.8 Hz), 2.85–2.92 (m, 1H), 3.01–3.16 (m, 6H), 3.22–3.28 (br s, 2H), 3.48–3.67 (m, 2H), 3.77–3.86 (m, 1H), 4.18–4.24 (m, 1H), 4.28 (d, 1H, *J* 9.5 Hz), 4.31–4.39 (m, 1H), 4.42–4.54 (m, 2H), 6.57–6.65 (m, 2H), 6.65–7.11 (br s, 2H, interfering with the next listed signal), 6.97–7.01 (m, 2H), 7.11–7.46 (br s, 2H), 7.50 (s, 1H), 7.55–7.67 (m, 2H), 7.88 (t, 1H, *J* 5.6 Hz), 7.98 (d, 1H, *J* 8.0 Hz), 8.04–8.28 (m, 4H), 8.29–8.56 (m, 2H), 8.59–8.73 (d, 1H, *J* 7.4 Hz), 8.99–9.12 (br s, 1H), 9.12–9.23 (m, 1H), 9.89–10.15 (br s, 1H), 12.35–12.61 (br s, 1H). HRMS (ESI): *m/z* [*M* + 2H]^2+^ calcd. for [C_48_H_80_N_14_O_10_]^2+^ 506.3085, found 506.3097. RP‐HPLC (220 nm): 96% (*t*_R_ = 8.5 min, *k* = 10.2). C_48_H_78_N_14_O_10_ · C_6_H_3_F_9_O_6_ (1011.24 + 342.07).

### *N*^α^-methyl-{*N*^ω^-[*N*-(4-pent-4-ynoylaminobutyl)aminocarbonyl]}Arg-Arg-Pro-Tyr-2-*tert*-butyl-Gly-Leu-OH tris(hydrotrifluoroacetate) (19)

A solution of **17** (1.96 mg, 10.1 μmol) in anhydrous DMF was added to a stirred solution of compound **16** tetrakis(hydrotrifluoroacetate) (11.7 mg, 8.38 μmol) and DIPEA (11.6 μL, 67.1 µmol) in anhydrous DMF/NMP (75:25 v/v) (68.9 µL) and stirring was continued at rt for 75 min. The mixture was acidified by addition of 10% aq. TFA (67.1 µL) and the product was purified by preparative RP-HPLC (column: Kinetex-XB C18; gradient: 0–35 min: A2/B 92:8–57:43, *t*_R_ = 21 min). Lyophilization of the eluate afforded **19** as white fluffy solid (10.3 mg, 90%). ^1^H-NMR (600 MHz, DMSO-*d*_6_): δ 0.81–0.93 (m, 15H), 1.32–1.89 (m, 19H), 1.96- 2.04 (m, 1H), 2.22–2.26 (m, 2H), 2.33–2.36 (m, 2H), 2.46–2.48 (m, 3H), 2.63–2.72 (m, 1H), 2.84–2.93 (m, 1H), 3.02–3.12 (m, 6H), 3.24–3.27 (m, 2H), 3.55–3.63 (m, 2H), 3.76–3.86 (m, 1H), 4.17–4.25 (m, 1H), 4.26–4.30 (m, 1H), 4.32–4.41 (m, 1H), 4.42–4.60 (m, 2H), 6.55–6.65 (m, 2H), 6.65–7.17 (br s, 2H, interfering with the next listed signal), 6.97–7.01 (m, 2H), 7.17–8.04 (m, 6H), 8.04–8.69 (m, 3H), 8.69–9.33 (m, 5H), 9.79–10.07 (m, 1H), 12.20–12.81 (br s, 1H). 1 exchangeable proton (NH, OH) of the presumably threefold protonated molecule could not be identified. HRMS (ESI): *m/z* [*M* + 2H]^2+^ calcd. for [C_49_H_82_N_14_O_10_]^2+^ 513.3164, found 513.3174. RP‐HPLC (220 nm): 99% (*t*_R_ = 8.8 min, *k* = 10.6). C_49_H_80_N_14_O_10_ · C_6_H_3_F_9_O_6_ (1025.27 + 342.07).

### H-(*N*^ω^-{*N*-[4-(3-{1-[6-deoxy-6-fluoro-β-D-glucopyranosyl]-1*H*-1,2,3-triazol-4-yl}propanoyl)aminobutyl]aminocarbonyl})Arg-Arg-Pro-Tyr-2-*tert*-butyl-Gly-Leu-OH tris(hydrotrifluoroacetate) (20)

Compound **20** was prepared from **18** (3.6 mg, 2.6 μmol) and **10** (2.2 mg, 10.6 μmol) using the procedure for the preparation of **11**. The product was purified by preparative RP-HPLC (column: Kinetex Biphenyl; gradient: 0–35 min: A1/B 90:10–62:38, *t*_R_ = 20 min). Lyophilization of the eluate afforded **20** as white fluffy solid (0.81 mg, 25%). HRMS (ESI): *m/z* [*M* + H]^+^ calcd. for [C_54_H_89_FN_17_O_14_]^+^ 1218.6753, found 1218.6737. RP‐HPLC (220 nm): > 99% (*t*_R_ = 7.6 min, *k* = 9.0). C_54_H_88_FN_17_O_14_ · C_6_H_3_F_9_O_6_ (1218.40 + 342.07).

### *N*^α^-methyl-(*N*^ω^-{*N*-[4-(3-{1-[6-deoxy-6-fluoro-β-D-glucopyranosyl]-1*H*-1,2,3-triazol-4-yl}propanoyl)aminobutyl]aminocarbonyl})Arg-Arg-Pro-Tyr-2-*tert*-butyl-Gly-Leu-OH tris(hydrotrifluoroacetate) (21)

A solution of **10** (1.4 mg, 6.75 μmol) in EtOH/PBS (1:9 v/v) (114 µL) and a solution of **19** (3.6 mg, 2.6 μmol) in NMP (108 µL) were combined. A 1 M solution of copper(II)sulfate pentahydrate (3.1 μL, 3.12 μmol) in PBS and a 1 M solution of sodium ascorbate (7.8 μL, 7.79 μmol) in PBS were added and the mixture was stirred at rt for 30 min. After acidification by addition of 10% aq. TFA (1 μL) the product was purified by preparative RP-HPLC (column: Gemini-NX C18; gradient: 0–35 min: A2/B 81:19–62:38, *t*_R_ = 11 min). Lyophilization of the eluate afforded **21** as white fluffy solid (3.2 mg, 79%). ^1^H-NMR (600 MHz, DMSO-*d*_6_): δ 0.80–0.96 (m, 15H), 1.34–1.44 (m, 4H), 1.44–1.90 (m, 14H), 1.93–2.06 (m, 1H), 2.40–2.49 (m, 5H), 2.63–2.71 (m, 1H), 2.80–2.93 (m, 3H), 2.97–3.18 (m, 6H), 3.20–3.28 (m, 3H), 3.40–3.44 (m, 1H), 3.51–3.65 (m, 2H), 3.67–3.85 (m, 3H), 4.17–4.24 (m, 1H), 4.28 (d, 1H, *J* 9.4 Hz), 4.33–4.40 (m, 1H), 4.43–4.65 (m, 4H), 5.38–5.48 (m, 2H), 5.57 (d, 1H, *J* 9.3 Hz), 6.58–6.63 (m, 2H), 6.63–7.16 (br s, 2H, interfering with the next listed signal), 6.97–7.01 (m, 2H), 7.16–7.78 (m, 4H), 7.78–8.11 (m, 3H), 8.12–8.59 (m, 3H), 8.61–9.36 (m, 4H), 9.88 (br s, 1H), 12.49 (br s, 1H). 3 exchangeable protons (NH, OH) of the threefold protonated molecule could not be identified. HRMS (ESI): *m/z* [*M* + H]^+^ calcd. for [C_55_H_91_FN_17_O_14_]^+^ 1232.6910, found 1232.6919. RP‐HPLC (220 nm): 98% (*t*_R_ = 7.6 min, *k* = 9.0). C_55_H_90_FN_17_O_14_ · C_6_H_3_F_9_O_6_ (1232.43 + 342.07).

### Radioligand competition binding assay

Radioligand competition binding experiments with [^3^H]UR-MK300 at hNTS_1_R expressing intact human HT-29 colon carcinoma cells (grown in antibiotic-free RPMI medium supplemented with 7.5% FCS) were performed at 23 ± 1 °C as described previously^3^. The latest determination of the *K*_d_ value of [^3^H]UR-MK300 in this assay yielded a *K*_d_ of 0.55 ± 0.03 nM (mean value ± SEM from two independent determinations performed in triplicate)^13^. Unspecific binding was subtracted from total binding to obtain specific binding. Data analysis was performed by plotting % specifically bound radioligand (100% = specifically bound radioligand in the absence of competitor) over log(concentration of competitor) followed by a four-parameter logistic fit (SigmaPlot 12.5, Systat Software). Resulting pIC_50_ values were converted to IC_50_ values and *K*_i_ values were calculated from the IC_50_ values according to the Cheng-Prusoff equation^33^ using a *K*_d_ value of 0.55 nM. The *K*_i_ values from individual experiments were transformed to p*K*_i_ values, followed by the calculation of mean p*K*_i_ values ± SEM.

### Investigation of the stability of 11–14, 16 and 18–21 in human plasma

The metabolic stabilities of **11**–**14**, **16** and **18**–**21** (*cf*. Table 2) were investigated in human blood plasma/PBS (136.9 mM NaCl, 2.68 mM KCl, 5.62 mM Na_2_HPO_4_, 1.09 mM NaH_2_PO_4_ and 1.47 mM KH_2_PO_4_) pH 7.4 (1:2, v/v) according to a described procedure^13^ with the following modifications: 5 mM stock solutions in EtOH/0.04% aq TFA (30:70 v/v) (**11**–**13**), MeCN/0.025% aq TFA (30:70 v/v) (**14**), MeCN/0.04% aq TFA (30:70 v/v) (**16**, **19** and **21**) or EtOH/H_2_O (40:60 v/v) (**18**, **20**) were used for the addition of the peptides to plasma/PBS (1:2 v/v). As the RP-HPLC purity of 1-Methyl-d-Trp (internal standard, IS) was < 95% (data not shown), the compound was purified by preparative HPLC to give a purity of > 99%. The concentration of the peptides in plasma/PBS (1:2 v/v) was 80 and 4 µM (recovery determination) or 100 µM (stability tests). The obtained recoveries and the recovery ratios (peptide/internal standard) are summarized in Supplementary Table S1. Data analysis was based on UV detection at 220 nm. Additionally, the stability of compound **21** was investigated in mouse plasma/PBS (1:2 v/v) using the same procedure as described above for human plasma. Mouse plasma was obtained by the collection of blood from anaesthetized mice via cardiac puncture using a syringe that was rinsed with sodium heparin (25,000 I.E., Ratiopharm, Ulm, Germany). The heparinized blood was transferred into a 2-mL reaction vessel, followed by centrifugation (1200 g, 4 °C, 10 min). The supernatants were pooled, centrifuged again (1200 g, 4 °C, 10 min), the plasma was aliquoted and stored at − 80 °C. The concentration of **21** in plasma/PBS (1:2 v/v) was 80 and 4 μM (recovery determination) or 100 μM (stability tests). The obtained recoveries and the recovery ratios (**21**/IS) are summarized in Supplementary Table S2. Data analysis was based on UV detection at 220 nm.

### Radiosynthesis of [^18^F]21

The ^18^F-labeled glycosyl azide 6-deoxy-6-[^18^F]fluoroglucosyl azide (**[**^**18**^**F]10**) was prepared and used for the following CuAAC as described previously with slight modifications^19^. In brief, [^18^F]fluoride was eluted from a Sep-Pak® Light (46 mg) Accell™ Plus QMA carbonate cartridge with a solution of Kryptofix® 2.2.2 (10 mg), potassium carbonate (0.1 M, 17.5 μL), KH_2_PO_4_ (0.1 M, 17.5 μL) in water (165 μL) and acetonitrile (800 μL). After azeotropic drying, the tosyl-precursor 2,3,4-tri-O-acetyl-6-O-tosylglucosyl azide (9 mg, 19 μmol) in anhydrous acetonitrile (450 μL) was added and the mixture was stirred at 85 °C for 5 min. The product 2,3,4-tri-O-acetyl-6-deoxy-6-[^18^F]fluoroglucosyl azide was isolated by semi-preparative HPLC (Kromasil C8, 125 × 8 mm, acetonitrile (0.1% TFA)/water (0.1% TFA) gradient from 30 to 70% in 30 min, *t*_R_ = 9.6 min) followed by SPE (Sep-Pak® light C18 cartridge, Waters). Starting from 1000 MBq [^18^F]fluoride, this procedure yielded 400–450 MBq of 2,3,4-tri-O-acetyl-6-deoxy-6-[^18^F]fluoroglucosyl azide (40–45% AY) after a total synthesis time of about 30 min. For subsequent CuAAc, deacetylation was achieved by treatment with NaOH (60 mM, 270 µL) for 5 min at 60 °C to afford **[**^**18**^**F]10**, and a mixture of Cu(OAc)_2_ (4 mM, 10 µL), THPTA (20 mM, 10 µL), sodium ascorbate (100 mM, 10 µL) and alkyne **19** (10 mM, 5 µL) in sodium phosphate buffer (0.5 M, pH 8.0, 270 µL) was added. After stirring for 10 min at 60 °C the radiochemical yield of **[**^**18**^**F]21** was 92% as determined by radio-HPLC (Chromolith RP-18e, 100 × 4.6 mm, acetonitrile (0.1% TFA)/water (0.1% TFA) gradient from 10 to 50% in 5 min, *t*_R_ = 2.97 min). The mixture was diluted with aqueous TFA (0.1%, 400 µL) and the product was isolated by semipreparative HPLC (Kromasil C8, 125 × 8 mm, acetonitrile (0.1% TFA)/water (0.1% TFA) gradient from 15 to 50% in 20 min, *t*_R_ = 7.2 min, see Supplementary Fig. S4). The product fraction was diluted with water (15 mL) and passed through an RP-18 cartridge (SepPak® light C18, Waters). The product was eluted with ethanol (1 mL). For all further experiments, the ethanol was evaporated in vacuo and the tracer was formulated with saline (0.9%). Starting from [^18^F]fluoride (600–1000 MBq), **[**^**18**^**F]21** was obtained in an AY of 20–23% (referred to [^18^F]fluoride) in a total synthesis time of 65–70 min in molar activities of 75–130 GBq/μmol (n = 5).

### In vitro characterization of [^18^F]21 by determination of logD_7.4_, stability in human serum and plasma, and binding to plasma proteins

All experiments were performed as described before^34^. In brief, the logD_7.4_ value was determined via an octanol/water partition assay and provided as mean value ± SD from three independent experiments, each performed in triplicates. The stability of **[**^**18**^**F]21** was determined by analytical radio-HPLC from human serum and human plasma samples (see Supplementary Fig. S5). The percentage of binding of **[**^**18**^**F]21** to human plasma proteins was determined by spin-column chromatography using MicroSpin™ G-50 columns (Cytiva, Amersham) and averaged in triplicate experiments.

### Tumor model

All mouse experiments were approved by the local animal protection authorities (Government of Central Franconia, Germany, no. 55.2-2532-2-279), were carried out in compliance with the ARRIVE guidelines and performed in accordance with the relevant institutional guidelines and EU regulations. Mice were maintained in groups in an IVC recovery unit (25 °C ± 1 °C, Tecniplast S.p.A, Italy) with autoclaved bedding, food, and water on a daily 12 h light/dark cycle. Female nude mice (8–10 weeks old, Crl:NMRI-Foxn1nu, Charles River) were used for animal studies and were kept under pathogen-free conditions at the Franz-Penzoldt-Zentrum (Friedrich-Alexander University Erlangen-Nürnberg). A cell suspension of HT-29 cells (2 × 10^6^) in PBS (100 µL) was injected in the upper back of each mouse. After 2 weeks the tumors were between 5 and 8 mm in diameter and the mice were used for biodistribution or PET studies.

### Biodistribution in HT-29 tumor-bearing nude mice

The tumor-bearing mice were anesthetized with O_2_/isoflurane (3–4% isoflurane, 0.8 L/min O_2_). Subsequently, the body weight and tumor size were determined, and the mice were laid on a heating pad (37 °C). About 1–3 MBq **[**^**18**^**F]21** (in 100 μL NaCl (0.9%) were injected via the tail vein. After 30, 60 or 90 min the mice (n = 4 for each time point) were euthanized by cervical dislocation under deep isoflurane anesthesia and the following organs/tissues of the mice were removed, weighed, and radioactivity counted in the γ-counter (Wallac Wizard, PerkinElmer): blood, lung, liver, heart, spleen, kidney, HT-29 tumor, brain, intestine, pancreas, duodenum, muscle, and femur. The results were presented as the percentage injected dose per gram organ (%ID/g), and tumor-to-organ ratios were calculated thereof. All measurements were corrected for decay.

### Small-animal PET imaging

The HT-29 tumor bearing mice (n = 4) were anesthetized using O_2_/isoflurane (3–4% isoflurane, 0.8 L/min O_2_) and laid on a heating pad (37 °C). Venous access was laid into the tail vein of the animals, and the cannula was fixed by an instant adhesive on the tail and the mice were transferred to the PET scanner. A dynamic PET scan was started from 0 to 60 min after injection of **[**^**18**^**F]21** (2.5–2.9 MBq, 100 μL). Blocking experiments were performed on the following day with the same mice. For this purpose, the mice were injected with radiotracer together with **3** (100 nmol per mouse) and also scanned from 0 to 60 min after injection. After iterative maximum a posteriori image reconstruction of the decay and attenuation-corrected images, regions of interest (ROIs) were drawn over the tumors using the software PMOD (PMOD Technologies LLC, Switzerland). The radioactivity concentration within the regions was obtained from the mean value within the multiple ROIs and then converted to percentage injected dose per gram organ (%ID/g).

### Stability in mouse blood

Two NMRI mice were anesthetized with isoflurane and injected with **[**^**18**^**F]21** (7–8 MBq) into the tail vein. One mouse was sacrificed by cervical dislocation after 10, the other mouse after 20 min, and approx. 100 μL blood were collected from the abdomen and transferred into Li-heparinized Microvettes® (100 LH, Sarstedt) and the Microvette® was centrifuged (2000 × g, 5 min). The supernatant was transferred in a reaction vial and the same volume of aqueous TFA (10%) was added. The vial was centrifuged (20,000 × g, 5 min) and a sample of the resulting supernatant (100 μL) was analyzed by radio-HPLC (Chromolith RP-18e, 100 × 4.6 mm, acetonitrile (0.1% TFA)/water (0.1% TFA) gradient from 10 to 50% in 5 min).

## Supplementary Information

Supplementary Information.

## Data Availability

All data reported in this manuscript are available upon reasonable request by contacting the corresponding author.

## References

[1] Maoret JJ (1994). Neurotensin receptor and its mRNA are expressed in many human colon cancer cell lines but not in normal colonic epithelium: Binding studies and RT-PCR experiments. Biochem. Biophys. Res. Commun..

[2] Reubi JC, Waser B, Friess H, Buchler M, Laissue J (1998). Neurotensin receptors: A new marker for human ductal pancreatic adenocarcinoma. Gut.

[3] Souaze F (2006). Expression of neurotensin and NT1 receptor in human breast cancer: A potential role in tumor progression. Cancer Res..

[4] Dupouy S (2011). The potential use of the neurotensin high affinity receptor 1 as a biomarker for cancer progression and as a component of personalized medicine in selective cancers. Biochimie.

[5] Wu Z, Martinez-Fong D, Tredaniel J, Forgez P (2012). Neurotensin and its high affinity receptor 1 as a potential pharmacological target in cancer therapy. Front. Endocrinol..

[6] Körner M, Waser B, Strobel O, Buchler M, Reubi JC (2015). Neurotensin receptors in pancreatic ductal carcinomas. EJNMMI Res..

[7] Binder EB, Kinkead B, Owens MJ, Nemeroff CB (2001). Neurotensin and dopamine interactions. Pharmacol. Rev..

[8] Ferraro L (2014). Neurotensin NTS1-dopamine D2 receptor-receptor interactions in putative receptor heteromers: Relevance for Parkinson's disease and schizophrenia. Curr. Protein Pept. Sci..

[9] Rostene WH, Alexander MJ (1997). Neurotensin and neuroendocrine regulation. Front. Neuroendocrinol..

[10] Carraway R, Leeman SE (1973). The isolation of a new hypotensive peptide, neurotensin, from bovine hypothalami. J. Biol. Chem..

[11] Maschauer S, Prante O (2018). Radiopharmaceuticals for imaging and endoradiotherapy of neurotensin receptor-positive tumors. J. Label. Comp. Radiopharm..

[12] Bruehlmeier M (2002). Stabilization of neurotensin analogues: Effect on peptide catabolism, biodistribution and tumor binding. Nucl. Med. Biol..

[13] Schindler L, Bernhardt G, Keller M (2019). Modifications at Arg and Ile give neurotensin(8–13) derivatives with high stability and retained NTS1 receptor affinity. ACS Med. Chem. Lett..

[14] Maschauer S, Einsiedel J, Hübner H, Gmeiner P, Prante O (2016). ^18^F- and ^68^Ga-labeled neurotensin peptides for pet imaging of neurotensin receptor 1. J. Med. Chem..

[15] Maschauer S (2010). Labeling and glycosylation of peptides using click chemistry: A general approach to ^18^F-glycopeptides as effective imaging probes for positron emission tomography. Angew. Chem. Int. Ed..

[16] Maschauer S (2010). Synthesis of a ^68^Ga-labeled peptoid-peptide hybrid for imaging of neurotensin receptor expression in vivo. ACS Med. Chem. Lett..

[17] Alshoukr F (2009). Novel neurotensin analogues for radioisotope targeting to neurotensin receptor-positive tumors. Bioconjug. Chem..

[18] Keller M (2020). Fluorescence labeling of neurotensin(8–13) via arginine residues gives molecular tools with high receptor affinity. ACS Med. Chem. Lett..

[19] Maschauer S, Haubner R, Kuwert T, Prante O (2014). ^18^F-glyco-RGD peptides for PET imaging of integrin expression: Efficient radiosynthesis by click chemistry and modulation of biodistribution by glycosylation. Mol. Pharm..

[20] Potemkin R, Strauch B, Kuwert T, Prante O, Maschauer S (2020). Development of ^18^F-fluoroglycosylated PSMA-ligands with improved renal clearance behavior. Mol. Pharm..

[21] Apostol CR, Hay M, Polt R (2020). Glycopeptide drugs: A pharmacological dimension between "small molecules" and "biologics". Peptides.

[22] Moradi SV, Hussein WM, Varamini P, Simerska P, Toth I (2016). Glycosylation, an effective synthetic strategy to improve the bioavailability of therapeutic peptides. Chem. Sci..

[23] Keller M (2016). Mimicking of arginine by functionalized n(omega)-carbamoylated arginine as a new broadly applicable approach to labeled bioactive peptides: High affinity angiotensin, neuropeptide y, neuropeptide FF, and neurotensin receptor ligands as examples. J. Med. Chem..

[24] Spinnler K (2020). An alkyne-functionalized arginine for solid-phase synthesis enabling "bioorthogonal" peptide conjugation. ACS Med. Chem. Lett..

[25] Miller SC, Scanlan TS (1997). Site-selective n-methylation of peptides on solid support. J. Am. Chem. Soc..

[26] Eaton, B. & Gold, L. Parallel selex allowing for asymmetrical reactions in combinatorial chemistry. USA patent (1999, patent US5858660A).

[27] Toms J (2020). Targeting fibroblast activation protein: Radiosynthesis and preclinical evaluation of an ^18^F-labeled FAP inhibitor. J. Nucl. Med..

[28] Delanghe S, Delanghe JR, Speeckaert R, Van Biesen W, Speeckaert MM (2017). Mechanisms and consequences of carbamoylation. Nat. Rev. Nephrol..

[29] Yadav SPS (2021). Mechanism of how carbamylation reduces albumin binding to FcRn contributing to increased vascular clearance. Am. J. Physiol. Renal Physiol..

[30] Wang M (2018). Development of [^18^F]AlF-NOTA-NT as PET agents of neurotensin receptor-1 positive pancreatic cancer. Mol. Pharm..

[31] Landau BR (2007). 6-fluoro-6-deoxy-d-glucose as a tracer of glucose transport. Am. J. Physiol. Endocrinol. Metab..

[32] Shinde SS, Maschauer S, Prante O (2021). Sweetening pharmaceutical radiochemistry by ^18^F-fluoroglycosylation: Recent progress and future prospects. Pharmaceuticals (Basel).

[33] Cheng Y, Prusoff WH (1973). Relationship between the inhibition constant (K_i_) and the concentration of inhibitor which causes 50 per cent inhibition (IC_50_) of an enzymatic reaction. Biochem. Pharmacol..

[34] Maschauer S (2019). F-18-labelled triazolyl-linked argininamides targeting the neuropeptide Y Y_1_R for PET imaging of mammary carcinoma. Sci. Rep..

