# Supplementary material for: Neurotensin analogs by fluoroglycosylation at Nω-carbamoylated arginines for PET imaging of NTS1-positive tumors
Source: Sci Rep. 2022 Sep 2;12:15028. doi: 10.1038/s41598-022-19296-0 (PMC9440028; doi:10.1038/s41598-022-19296-0)
Supplement: Supplementary file 1 — Supplementary Information. [file 41598_2022_19296_MOESM1_ESM.pdf]

Supplementary Information

Neurotensin analogs by fluoroglycosylation at N^{ω}-carbamoylated arginines for PET imaging of NTS1-positive tumors

Lisa Schindler[1], Katrin Wohlfahrt[1,‡], Lara Gluhacevic von Krüchten[1], Olaf Prante[2], Max Keller[1,*], Simone Maschauer[2,*]

[1]Institute of Pharmacy, Faculty of Chemistry and Pharmacy, University of Regensburg, Universitätsstrasse 31, 93053 Regensburg, Germany

[2]Department of Nuclear Medicine, Molecular Imaging and Radiochemistry, Friedrich-Alexander-Universität Erlangen-Nürnberg (FAU), Schwabachanlage 12, 91054 Erlangen, Germany

[‡]current address: Hennig Arzneimittel GmbH & Co KG, Liebigstr. 1-2, 65439 Flörsheim am Main

[*]Corresponding authors

Content **Pages**

1. General S2
2. Tables S1 and S2 and Figures S1 and S2 S4-S6
3. Figures S3-S5 and Table S3:
   - Figure S3. Structures of the NT(8-13)-derived NTS1 PET ligands **[^{18}F]4**[2], **[^{18}F]21**, and the NT(6-13)-derived [^{18}F]AlF-NOTA-NT[3] for comparison S7
   - Figure S4. Semipreparative HPLC of **[^{18}F]21** and analytical HPLC of **21** and **19** S8
   - Figure S5: Radiochemical purity, stability *in vitro* and *ex vivo* of **[^{18}F]21** S9
   - Table S3: Biodistribution data of **[^{18}F]21** in HT-29 tumor-bearing mice S10
4. RP-HPLC analyses of compounds **7-9, 11-14, 16** and **18-21** S11-S14
5. ^{1}H-NMR spectra of compounds **7-9**, **11-14**, **16-19** and **21**, and ^{13}C-NMR spectra of compounds **7-9**, **14**, **16** and **17** S15-S23
6. References S24

1. General

If not otherwise stated, solvents and buffer components, purchased from commercial suppliers, were of analytical grade. Gradient grade MeOH for HPLC was obtained from Merck (Darmstadt, Germany) and gradient grade acetonitrile for HPLC was from Sigma-Aldrich (Taufkirchen, Germany). Diethyl ether was from Fisher Chemicals (Loughborough, United Kingdom), EtOAc was from VWR Chemicals (Ismaning, Germany), and *N*,*N*-Diisopropylethylamine (DIPEA, 99%) was from ABCR (Karlsruhe, Germany). Anhydrous *N*,*N*-Dimethylformamide (DMF) (99.8%), n-hexane, 1,1,1,3,3,3-hexafluoro-2-propanol (HFIP), 7-methyl-1,5,7-triazabicyclo[4.4.0]dec-5-en (MTBD), methyl-4-nitrobenzene-sulfonate (**15**), 2-mercaptoethanol, *N*-hydroxysuccinimide, *N*,*N'*-dicyclohexylcarbodiimide (DCC), sodium L-ascorbate and 1-methyl-D-Trp were purchased from Sigma-Aldrich. DMF (for peptide synthesis, packed under nitrogen, code D/3848/PB17), 1-methyl-2-pyrrolidone (NMP) (for peptide synthesis, nitrogen flushed), anhydrous NMP (99.5%), CH_2Cl_2 and 1-hydroxy-1*H*-benzotriazole (HOBt) hydrate were obtained from Acros Organics/Fisher Scientific (Nidderau, Germany). Trifluoroacetic acid and absolute EtOH were purchased from Honeywell (Seelze, Germany). 4-Pentynoic acid, collidine, 2-nitrobenzenesulfonylchloride and 1,8-diazabicyclo[5.4.0]undec-7-ene (DBU) were from Alfa Aesar/ThermoFisher (Heysham, Great Britain). Piperidine and *N*,*N*,*N'*,*N'*-tetramethyl-*O*-(1*H*-benzotriazole-1-yl)-uronium hexafluorophosphate (HBTU) were from Iris Biotech (Marktredwitz, Germany). Deuterated solvents were obtained from Deutero (Kastellaun, Germany). Bovine serum albumin (BSA) was purchased from Serva (Heidelberg, Germany). Ammonium acetate (98%) and copper(II)sulfate pentahydrate were from Merck. H-Leu-2-ClTrt resin (loading: 0.79 mmol/g), Fmoc-N-Me-Arg(Pbf)-OH, Fmoc-Pro-OH, Fmoc-Tle-OH (Fmoc-α-*tert*-butylglycine)) were purchased from Merck Biosciences (Schwalbach am Taunus, Germany). Fmoc-Arg(Pbf)-OH and Fmoc-Tyr(*t*Bu)-OH were obtained from Iris Biotech. Peptide **1** (tris(hydrotrifluoroacetate)) was purchased from SynPeptide (Shanghai, China).

Succinimidyl pentynoate (**17**)[4] and 6-deoxy-6-fluoro-*β*-D-glucosyl azide (**10**)[5] were prepared according to described procedures. Millipore water was used throughout for the preparation of buffers, stock solutions and HPLC eluents. 1.5- and 2-mL polypropylene reaction vessels with screw cap (in the following referred to as "reaction vessel with screw cap") from Süd-Laborbedarf (Gauting, Germany) were used for the preparation and storage of stock solutions, and for small-scale reactions. 1.5- or 2-mL polypropylene reaction vessels (in the following referred to as "reaction vessel") from Sarstedt (Nümbrecht, Germany) were used for the preparation of diluted solutions, and for the investigation of stabilities in plasma. For the evaporation of solvents in 1.5- or 2-mL reaction vessels, a Savant Speed-Vac Plus SC110A vacuum concentrator (Thermo Fischer Scientific, Waltham, MA) was used. NMR spectra were recorded on a Bruker Avance 600 instrument (^{1}H: 600 MHz, T = 300 K, ^{13}C: 151 MHz, T = 300 K), a Bruker Avance 400 instrument (^{1}H: 400 MHz, T = 300 K, ^{13}C: 101 MHz, T = 300 K) or a Bruker Avance 300 instrument (^{1}H: 300 MHz, T = 300 K, ^{13}C: 75 MHz, T = 300 K) (Bruker, Karlsruhe, Germany). The spectra were calibrated based on the solvent residual peaks (^{1}H-NMR: $CDCl_3$: δ = 7.26 ppm, DMSO-d_6: δ = 2.50 ppm; ^{13}C-NMR: DMSO-d_6: δ = 39.50 ppm). ^{1}H-NMR data are reported as follows: chemical shift δ in ppm (multiplicity (s = singlet, d = doublet, t = triplet, q = quartet, m = multiplet, br s = broad singlet), integral, coupling constant *J* in Hz). Thin layer chromatography was performed on Merck silica gel 60 F254 TLC aluminum plates. High resolution mass spectra (HRMS) were acquired with an Agilent 6540 UHD Accurate-Mass Q-TOF LC/MS system coupled to an Agilent 1290 HPLC system (Agilent Technologies, Santa Clara, CA), using an ESI source. Analyses were performed using the following LC method: column: Luna Omega C18, 1.6 µm, 50 × 2.1 mm (Phenomenex, Aschaffenburg, Germany), column temperature: 40 °C, flow: 0.6 mL/min, solvent/linear

gradient: 0-4 min: 0.1% aq HCOOH/0.1% HCOOH in MeCN 95:5-2:98, 4-5 min: 2:98. Preparative HPLC was performed with a system from Knauer (Berlin, Germany) consisting of two K-1800 pumps and a K-2001 detector. A Kinetex-XB C18, 5 µm, 250 mm × 21 mm (Phenomenex), a Kinetex Biphenyl, 5 µm, 250 mm × 21 mm (Phenomenex) or a Gemini-NX C18, 5 µm, 250 mm × 21 mm (Phenomenex) served as RP-columns at a flow rate of 20 mL/min. Mixtures of 0.1% aq TFA (A1) and acetonitrile (B), or 0.2% aq TFA (A2) and B were used as mobile phase. A detection wavelength of 220 nm was used throughout. Collected fractions were lyophilized using an Alpha 2-4 LD apparatus (Martin Christ, Osterode am Harz, Germany) or a Scanvac CoolSafe 100-9 freeze-dryer (Labogene, Allerød, Denmark) both equipped with a vacuubrand RZ 6 rotary vane vacuum pump. Analytical HPLC analysis of compounds **7**-**9**, **11**-**14**, **16** and **18**-**21** was performed with a system from Agilent Technologies consisting of a 1290 Infinity binary pump equipped with a degasser, a 1290 Infinity Autosampler, a 1290 Infinity Thermostated Column Compartment, a 1260 Infinity Diode Array Detector and a 1260 Infinity Fluorescence Detector. A Kinetex-XB C18, 2.5 µm, 100 × 3 mm (Phenomenex) served as stationary phase at a flow rate of 0.6 mL/min. The oven temperature was set to 25 °C. Mixtures of 0.04% aq TFA (A3) and B were used as mobile phase. The following linear gradient was applied: 0-12 min: A3/B 90:10-70:30, 12-16 min: 70:30-5:95, 16-20 min: 5:95. The injection volume was 20 µL. UV detection was performed at 220 nm and fluorescence detection at 275/305 nm. Retention (capacity) factors k were calculated from the retention times t_R according to $k = (t_R - t_0)/t_0$ (t_0 = dead time). Synthesized peptides were characterized by ^{1}H- and ^{13}C-NMR spectroscopy, HRMS, and RP-HPLC analysis. Additionally, ^{1}H-COSY NMR spectra were acquired of peptides **7**-**9**, **14**, **16**, **19** and **21**. The purity of all final compounds, determined by RP-HPLC (220 nm), was ≥ 96%.

Annotation concerning the ^{1}H-NMR spectra (solvent: DMSO-d_6): in order to allow an integration of the signals interfering with the broad water signal at ca 3.5 ppm, ^{1}H-NMR spectra were additionally recorded in DMSO-d_6/D_2O (10:1 or 4:1 v/v) (spectra and data not shown).

Radio-HPLC was performed on an Agilent 1100 system (Agilent Technologies) with a quaternary pump and variable wavelength detector and radio-HPLC detector HERM LB 500 (Berthold Technologies, Germany). No-carrier-added [^{18}F]fluoride was produced through the ^{18}O(p,n)^{18}F reaction on a PETtrace 800 cyclotron (General Electric, Uppsala, Sweden) using H_2[^{18}O]O as the target at the Universitätsklinikum Würzburg (Klinik und Poliklinik für Nuklearmedizin, Experimentelle Nuklearmedizin, Radiopharmazie/PET-Zentrum, Prof. Samuel Samnick) and transported by road to Erlangen.

2. Tables S1 and S2 and Figures S1 and S2

Table S1. Recoveries of peptides **11-14, 16** and **18-21** from human plasma/PBS (1:2 v/v) and ratios of peptide-recovery over recovery of IS.

Compd.	Peptide concentration 80 μM			Peptide concentration 4 μM		
	recovery peptide (%)[a]	recovery IS (%)[a]	ratio[b]	recovery peptide (%)[a]	recovery IS (%)[a]	ratio[b]
**11**	94	107	0.88	94	99	0.95
	91	99	0.92	89	100	0.89
	97	109	0.89	94	102	0.92
	89	97	0.92	91	99	0.92
	92	101	0.91	104	108	0.96
			(0.90 ± 0.01)			(0.93 ± 0.01)
**12**	91	106	0.85	92	96	0.96
	93	107	0.87	116	105	1.11
	94	110	0.85	103	99	1.03
	92	107	0.86	110	100	1.10
			(0.86 ± 0.01)			(1.05 ± 0.03)
**13**	86	104	0.82	103	101	1.03
	88	103	0.85	104	106	0.98
	92	104	0.89	104	98	1.06
	89	99	0.90	93	89	1.05
			(0.86 ± 0.02)			(1.03 ± 0.02)
**14**	85	104	0.82	88	105	0.84
	84	106	0.79	96	110	0.87
	84	103	0.82	94	109	0.87
	88	107	0.82	94	106	0.89
				88	100	0.88
			(0.81 ± 0.01)			(0.87 ± 0.01)
**16**	91	104	0.87	91	97	0.93
	88	104	0.85	91	99	0.92
	88	105	0.84	98	106	0.93
	94	110	0.85	101	109	0.93
			(0.85 ± 0.01)			(0.93 ± 0.01)
**18**	95	104	0.91	102	103	0.99
	111	120	0.93	119	122	0.97
	99	107	0.92	104	98	1.06
	98	110	0.89	112	112	1.00
	109	120	0.91	98	103	0.95
			(0.91 ± 0.01)			(0.99 ± 0.02)
**19**	93	101	0.92	106	94	1.12
	93	100	0.93	100	94	1.07
	96	101	0.95	97	88	1.10
	101	106	0.96	105	100	1.05
			(0.94 ± 0.01)			(1.09 ± 0.02)
**20**	86	97	0.88	109	100	1.09
	94	103	0.91	109	103	1.06
	90	99	0.91	110	108	1.02
	89	96	0.92	113	109	1.03
				115	107	1.07
			(0.91 ± 0.01)			(1.05 ± 0.01)
**21**	96	104	0.92	102	103	0.99
	99	106	0.93	112	107	1.05
	103	111	0.92	110	109	1.01
	104	112	0.93	119	115	1.03
				108	109	0.99
			(0.93 ± 0.01)			(1.01 ± 0.01)

[a]Recoveries of the peptides and of IS from human plasma/PBS (1:2 v/v) using a peptide concentration of 80 μM or 4 μM and an IS concentration of 10 μM (four or five independent experiments). [b]Ratios of peptide recovery over recovery of the IS calculated for individual experiments, as well as mean recovery ratios ± SEM (given in parenthesis). Note: When the remaining intact peptide concentration in plasma was >20 μM, recovery ratios based on the 80 μM peptide concentrations were used to calculate peptide recoveries of the plasma stability samples. When the remaining intact peptide concentration was <20 μM, recovery ratios based on the 4 μM peptide concentrations were used to calculate peptide recoveries of the plasma stability samples.

Table S2. Recoveries of potential PET ligand **21** from mouse plasma/PBS (1:2 v/v) and ratios of peptide-recovery over recovery of IS.

Compd.	Peptide concentration 80 µM			Peptide concentration 4 µM		
	recovery peptide (%)[a]	recovery IS (%)[a]	ratio[b]	recovery peptide (%)[a]	recovery IS (%)[a]	ratio[b]
**21**	95	103	0.92	105	105	1.00
	92	102	0.90	101	104	0.98
	96	105	0.91	107	105	1.02
	93	100	0.93	107	104	1.03
				108	108	0.99
			(0.92 ± 0.01)			(1.00 ± 0.01)

[a]Recoveries of **21** and IS from mouse plasma/PBS (1:2 v/v) using a peptide concentration of 80 µM or 4 µM and an IS concentration of 10 µM (four or five independent experiments). [b]Ratios of peptide recovery over recovery of the IS calculated for individual experiments, as well as mean recovery ratios ± SEM (given in parenthesis). Note: As the remaining intact peptide concentration in plasma was >20 µM, recovery ratios based on the 80 µM peptide concentrations were used to calculate peptide recoveries of the plasma stability samples.

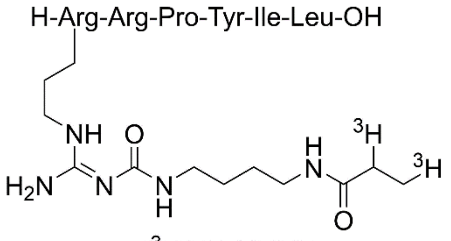

[3H]UR-MK300

Figure S1. Structure of the tritium-labeled NT(8-13)-derived radioligand [^{3}H]UR-MK300 used for NTS$_1$R competition binding studies (reported K_d = 0.51 nM).[1]

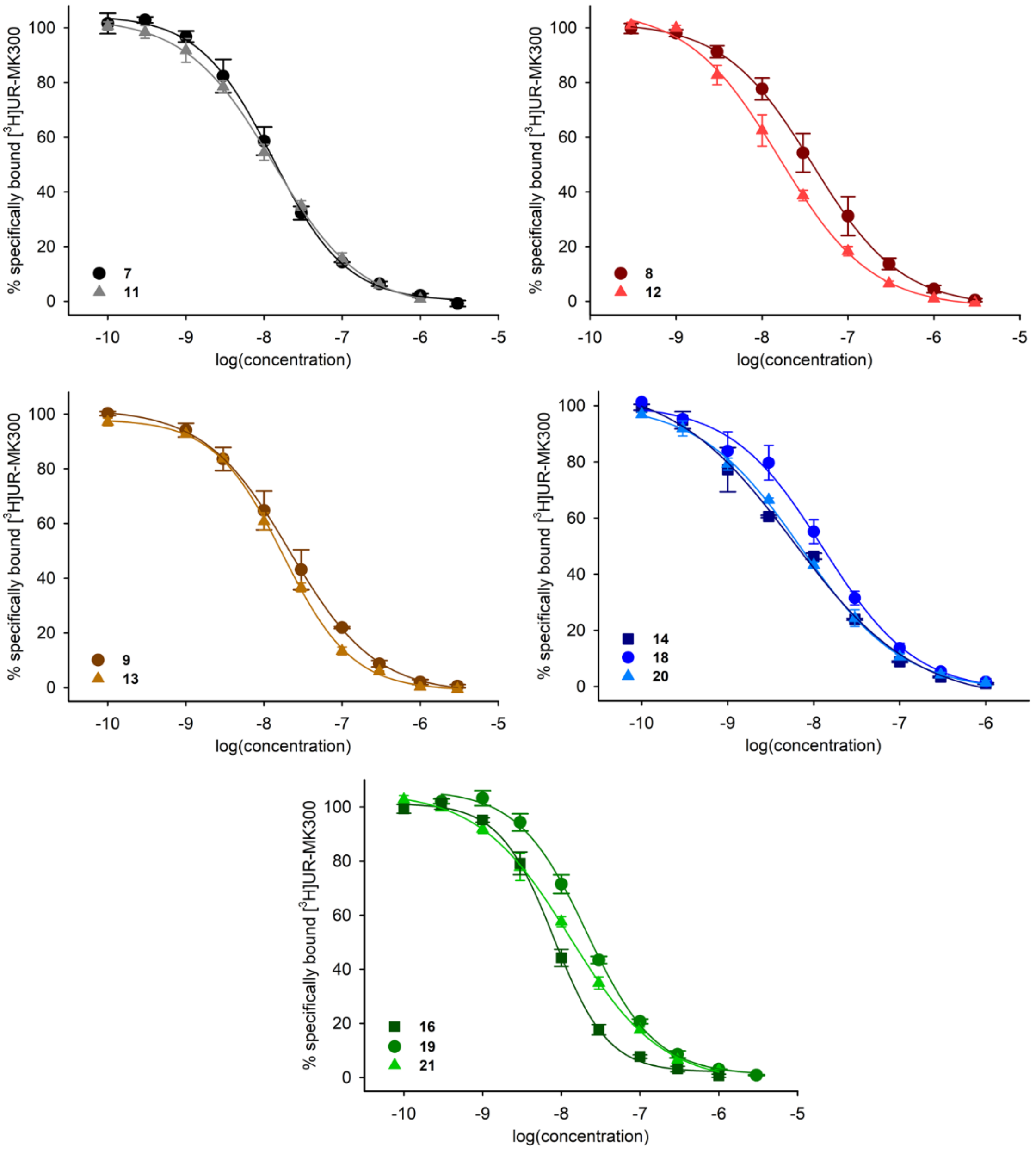

Figure S2. Radioligand displacement curves from competition binding experiments with [^{3}H]UR-MK300 (K_d = 0.55 nM, c = 1 nM) and **7-9**, **11-14**, **16** or **18-21** at intact hNTS$_1$R expressing HT-29 cells. Amino-functionalized precursor peptides are represented by squares, alkyne-functionalized peptides are represented by circles, and compounds conjugated to the sugar moiety are represented by triangles. Data represent mean values ± SEM from at least two independent experiments (performed in triplicate).

3. Figures S3-S5 and Table S3

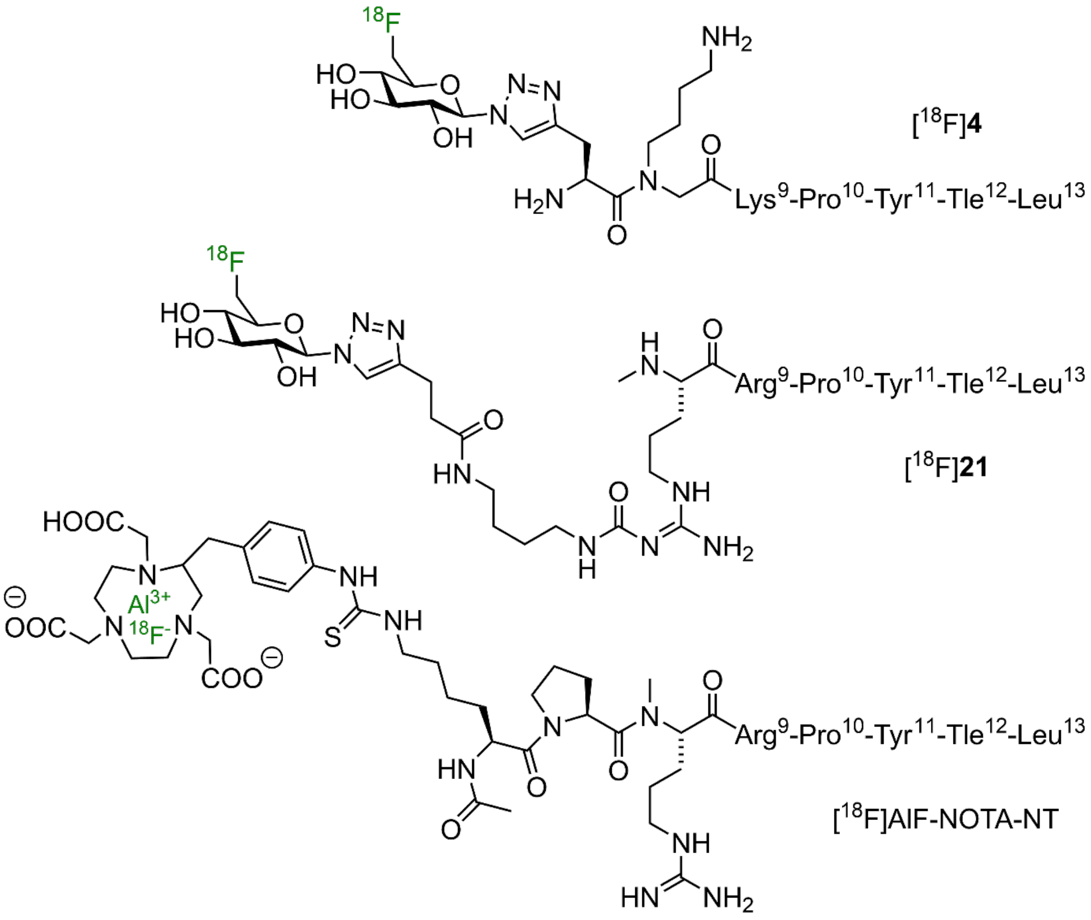

Figure S3. Structures of the NT(8-13)-derived NTS1 PET ligands [^{18}F]**4**[2] and [^{18}F]**21** (this work), both conjugated to an ^{18}F-labeled 6-deoxy-6-fluoroglucosyl moiety, and the NT(6-13)-derived [^{18}F]AlF-NOTA-NT,[3] containing $^{18}F^{-}$, non-covalently bound to Al^{3+} in the NOTA chelator.

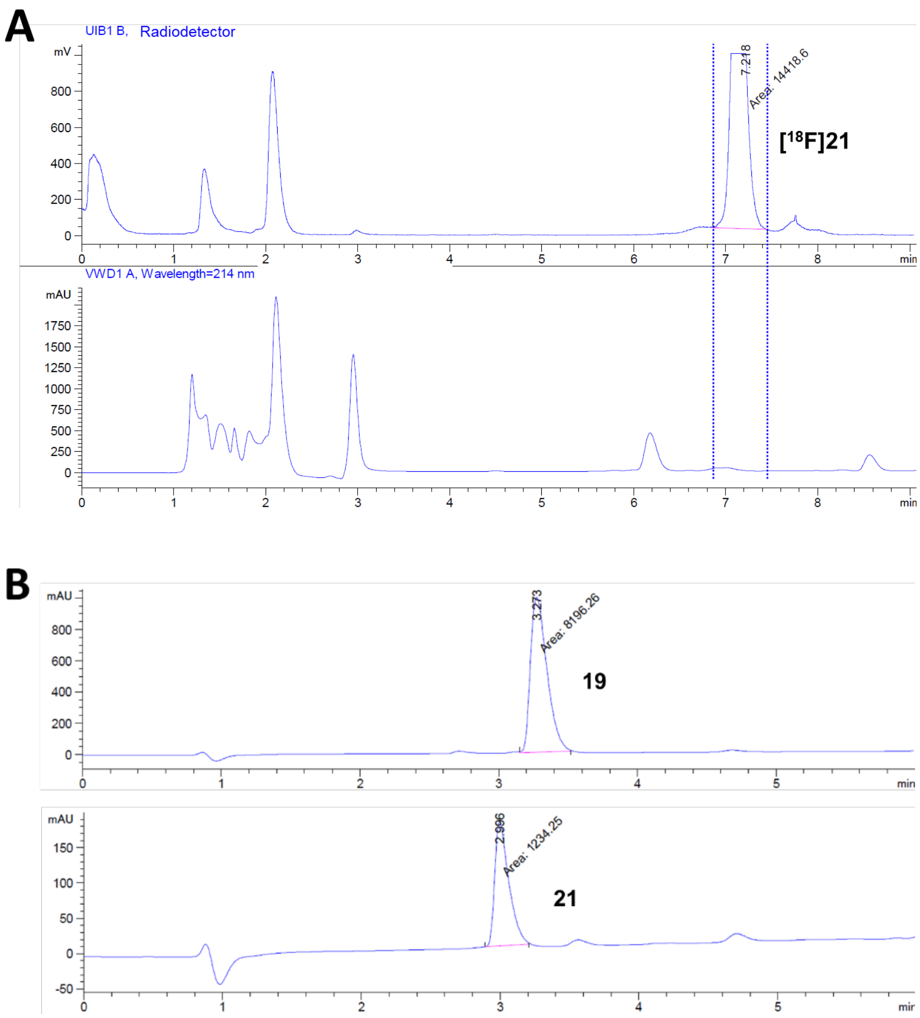

Figure S4. (**A**) Semipreparative HPLC run for isolation of **[^{18}F]21** (HPLC: Kromasil C8, 125 × 8 mm, acetonitrile (0.1% TFA)/water (0.1% TFA) gradient from 15 to 50% in 20 min, t_R (**[^{18}F]21**)= 7.2 min, note: t_R (**19**) > 9 min, not recorded). (**B**) HPLC analysis of glycopeptide **21** compared to the alkyne precursor **19** revealed that **19** is more lipophilic than **21** (HPLC: Chromolith RP-18, 100 × 4.6 mm, 4 mL/min, 10-50% acetonitrile (0.1% TFA) in water (0.1% TFA) in 5 min, detection at 214 nm, t_R (**21**) = 3.0 min, t_R (**19**) = 3.3 min).

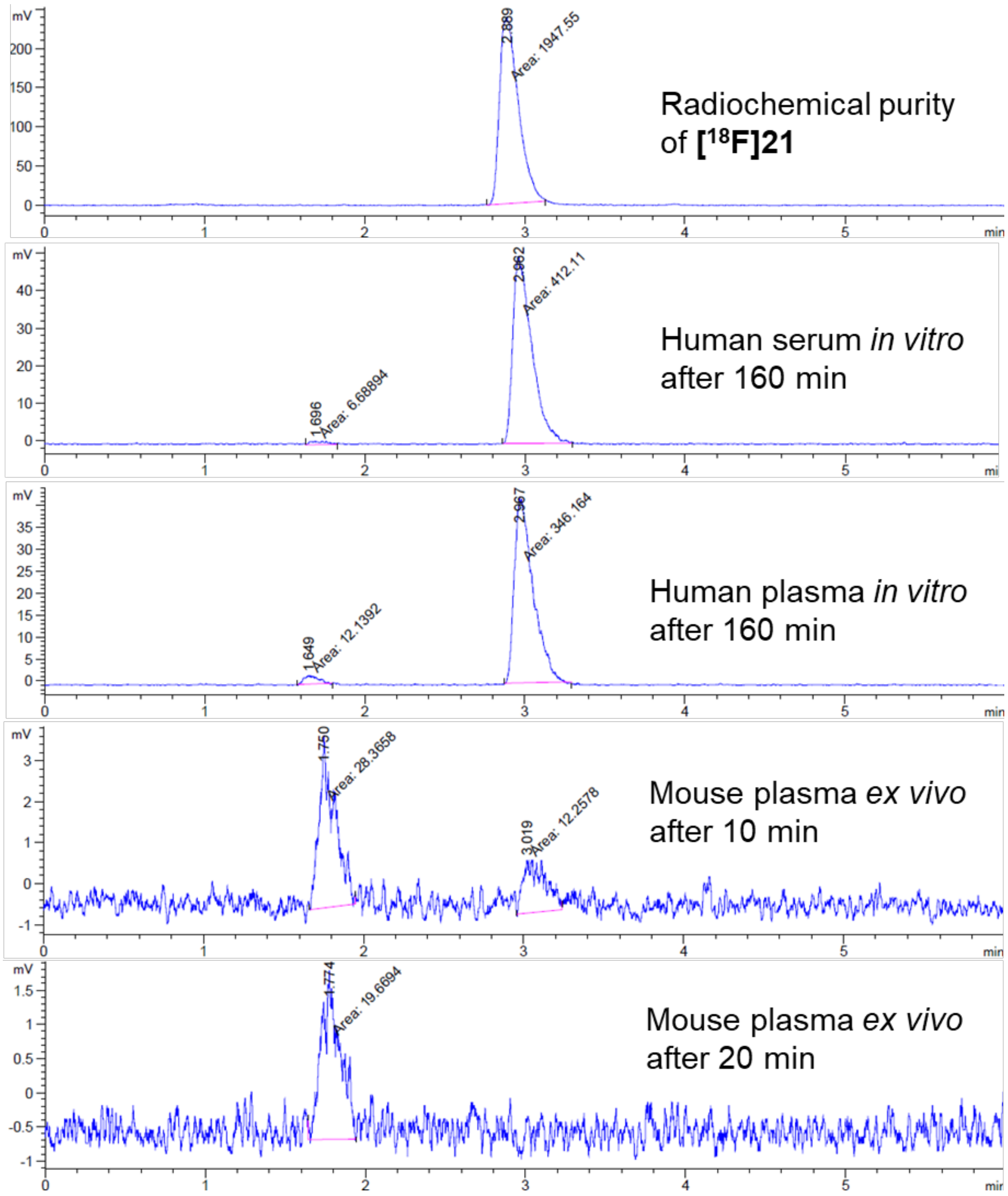

Figure S5. Radiochemical purity and stability *in vitro* and *ex vivo* (after i.v. injection) of **[^{18}F]21**. HPLC: Chromolith RP-18, 100 × 4.6 mm, 4 mL/min, 10-50% acetonitrile (0.1% TFA) in water (0.1% TFA) in 5 min.

Table S3. Biodistribution data (%ID/g) of **[^{18}F]21** in HT-29 tumor-bearing nude mice at 30, 60 and 90 min p.i. and tumor-to-tissue ratios calculated thereof from selected organs. Values are given as the mean ± standard deviation (n = 4).

	30 min	60 min	90 min
Blood	1.7 ± 1.1	0.4 ± 0.4	0.1 ± 0.05
Lung	1.5 ± 0.2	0.3 ± 0.2	0.3 ± 0.2
Liver	5.1 ± 0.5	4.1 ± 0.3	3.7 ± 0.6
Kidneys	16.0 ± 3.3	14.8 ± 3.0	11.9 ± 1.4
Heart	0.6 ± 0.1	0.2 ± 0.1	0.07 ± 0.04
Spleen	1.3 ± 0.3	1.0 ± 0.4	0.7 ± 0.1
Brain	0.1 ± 0.05	0.04 ± 0.02	0.04 ± 0.05
Muscle	2.6 ± 5.2	0.4 ± 0.5	0.08 ± 0.04
Femur	2.2 ± 3.9	0.7 ± 1.1	0.2 ± 0.02
HT-29 tumor	4.8 ± 1.0	2.1 ± 0.6	2.6 ± 0.3
Intestine	2.5 ± 1.5	1.8 ± 2.0	0.5 ± 0.5
Pancreas	0.6 ± 0.4	0.7 ± 1.1	0.2 ± 0.2
Duodenum	1.1 ± 0.3	1.4 ± 1.8	0.5 ± 0.3
Tumor/Blood	2.8	5.3	30.0
Tumor/Kidneys	0.3	0.1	0.2
Tumor/Muscle	7.6	6.0	32.6

4. RP-HPLC analyses of compounds 7-9, 11-14, 16 and 18-21

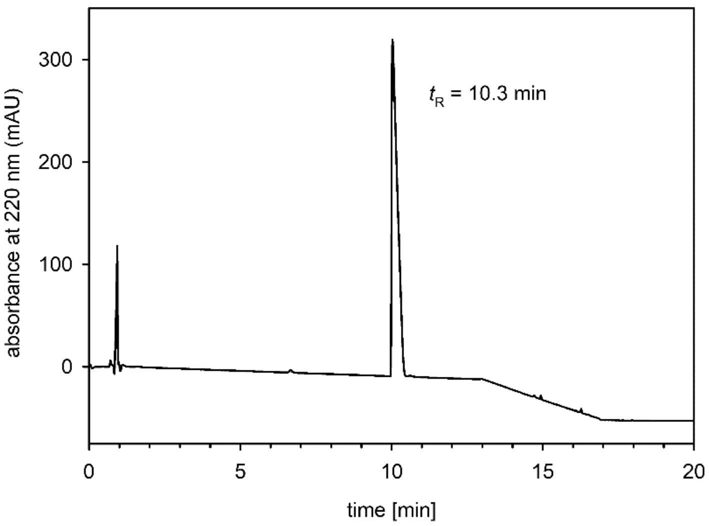

RP-HPLC analysis (purity control) of compound **7**

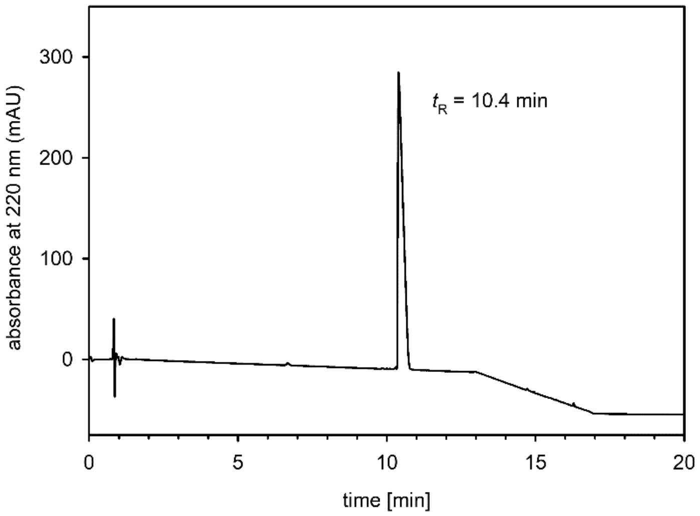

RP-HPLC analysis (purity control) of compound **8**

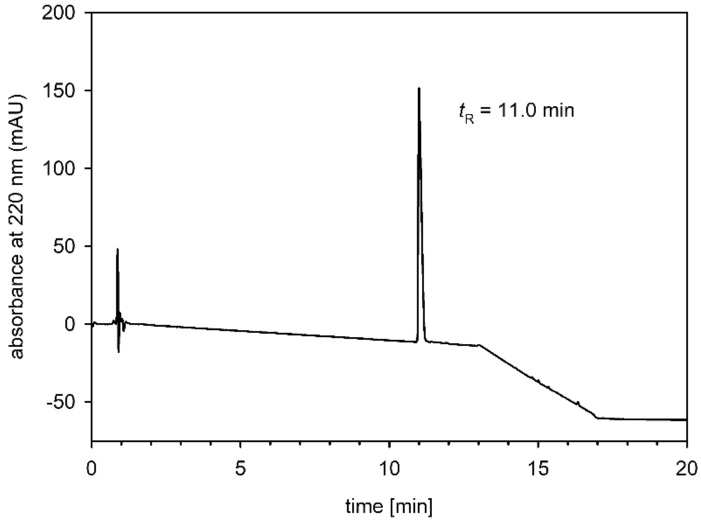

RP-HPLC analysis (purity control) of compound **9**

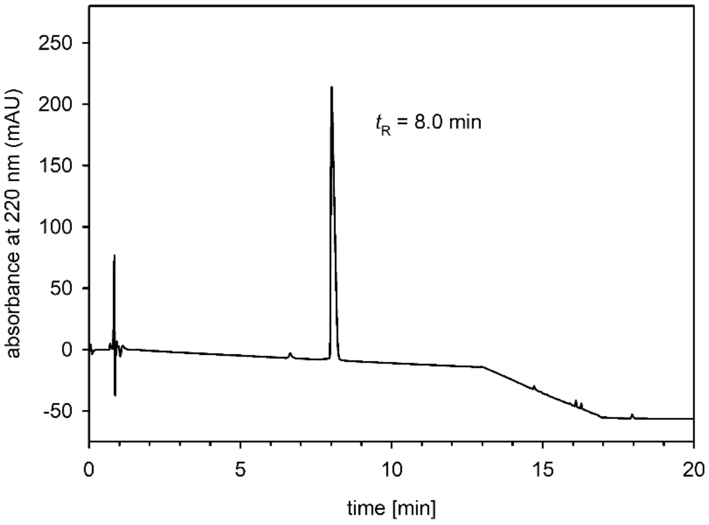

RP-HPLC analysis (purity control) of compound **11**

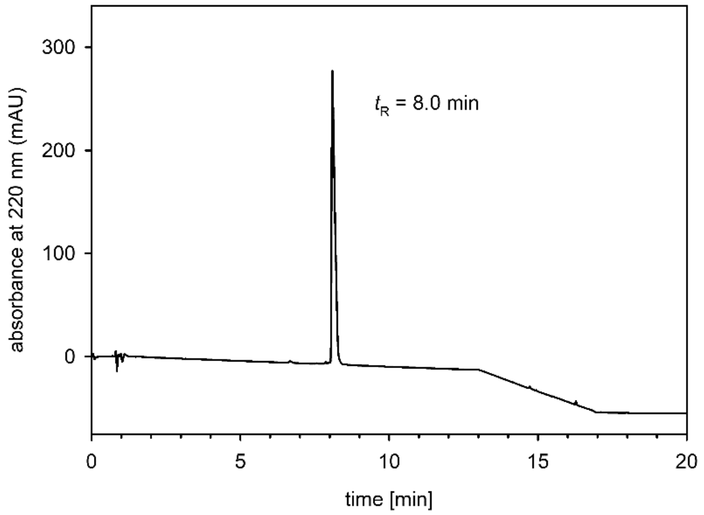

RP-HPLC analysis (purity control) of compound **12**

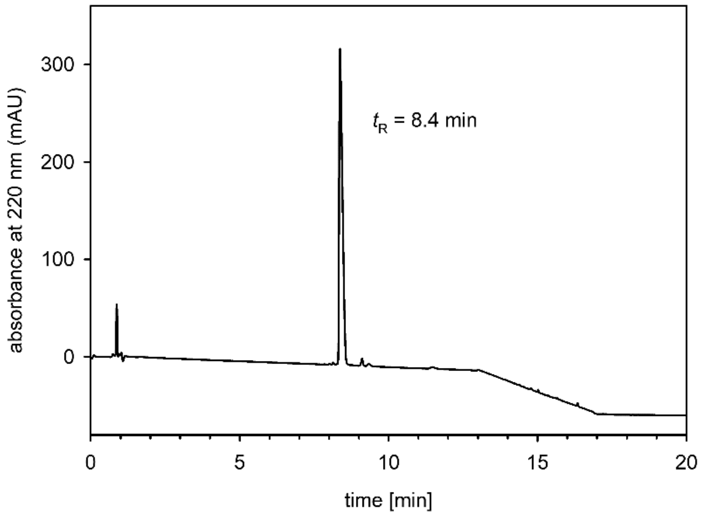

RP-HPLC analysis (purity control) of compound **13**

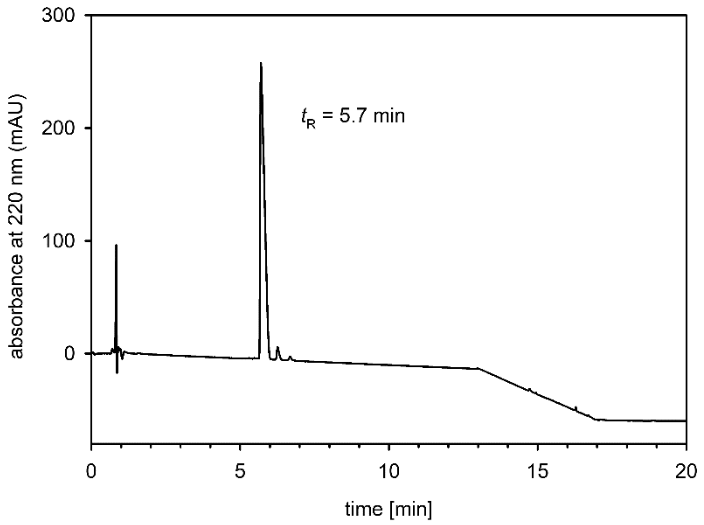

RP-HPLC analysis (purity control) of compound **14**

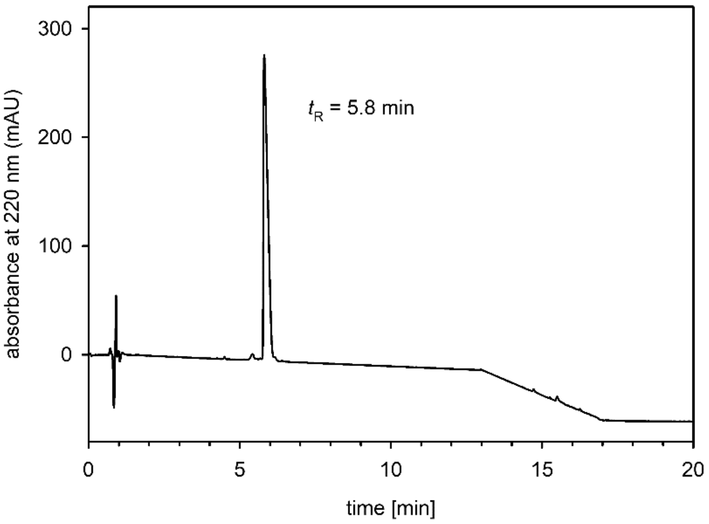

RP-HPLC analysis (purity control) of compound **16**

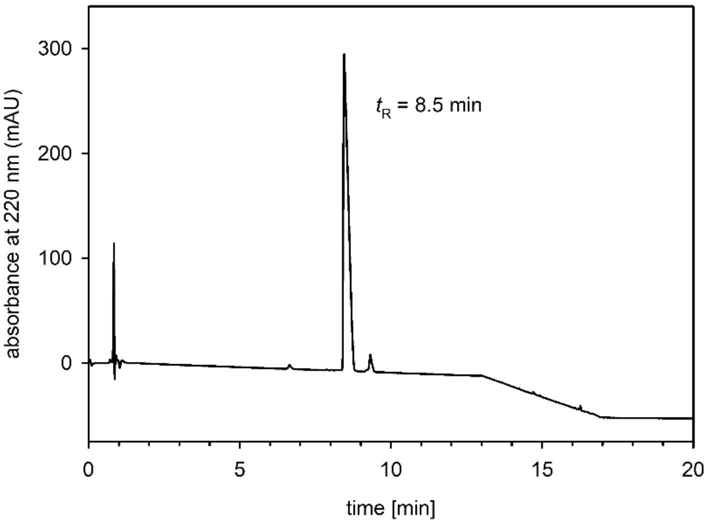

RP-HPLC analysis (purity control) of compound **18**

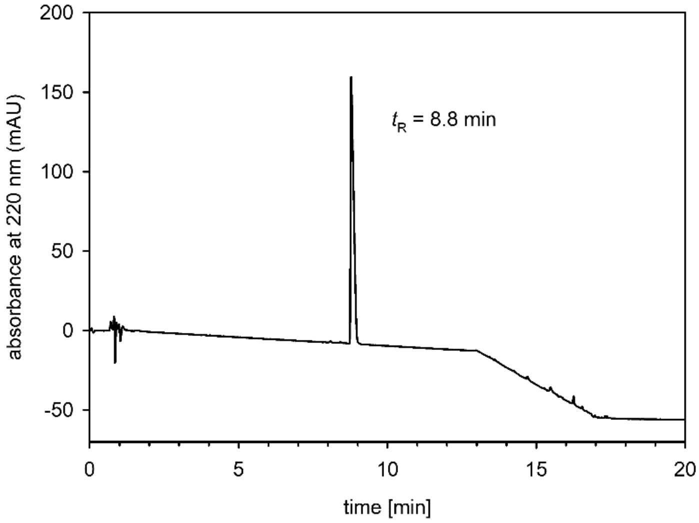

RP-HPLC analysis (purity control) of compound **19**

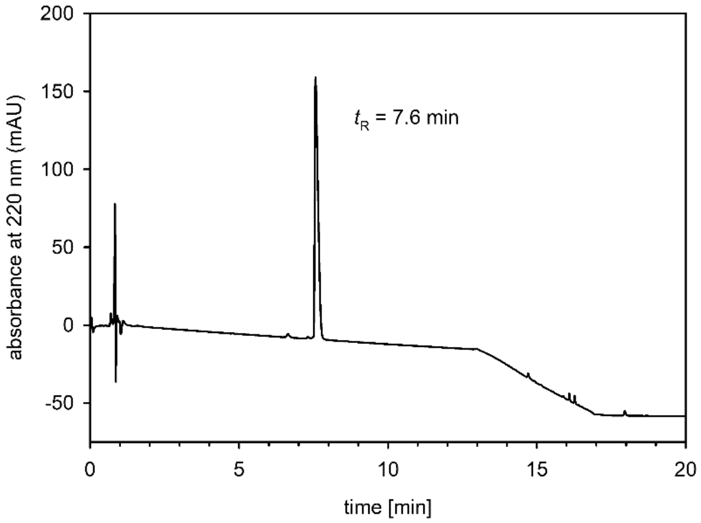

RP-HPLC analysis (purity control) of compound **20**

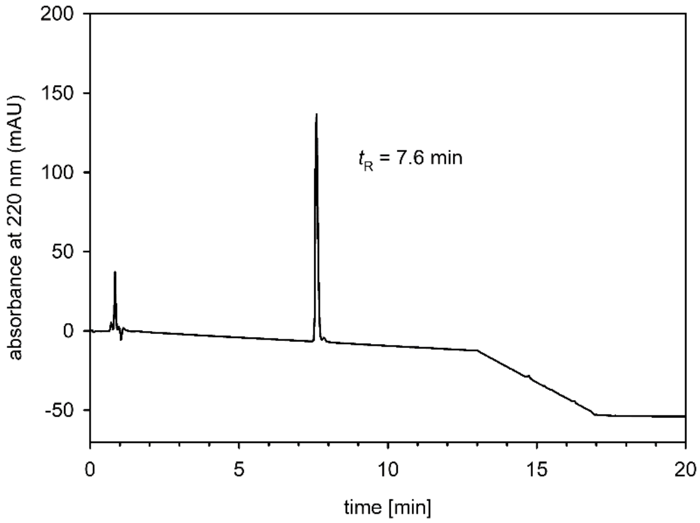

RP-HPLC analysis (purity control) of compound **21**

5. ^{1}H-NMR spectra of compounds 7-9, 11-14, 16, 18, 19 and 21, and ^{13}C-NMR spectra of compounds 7-9, 14, 16 and 17

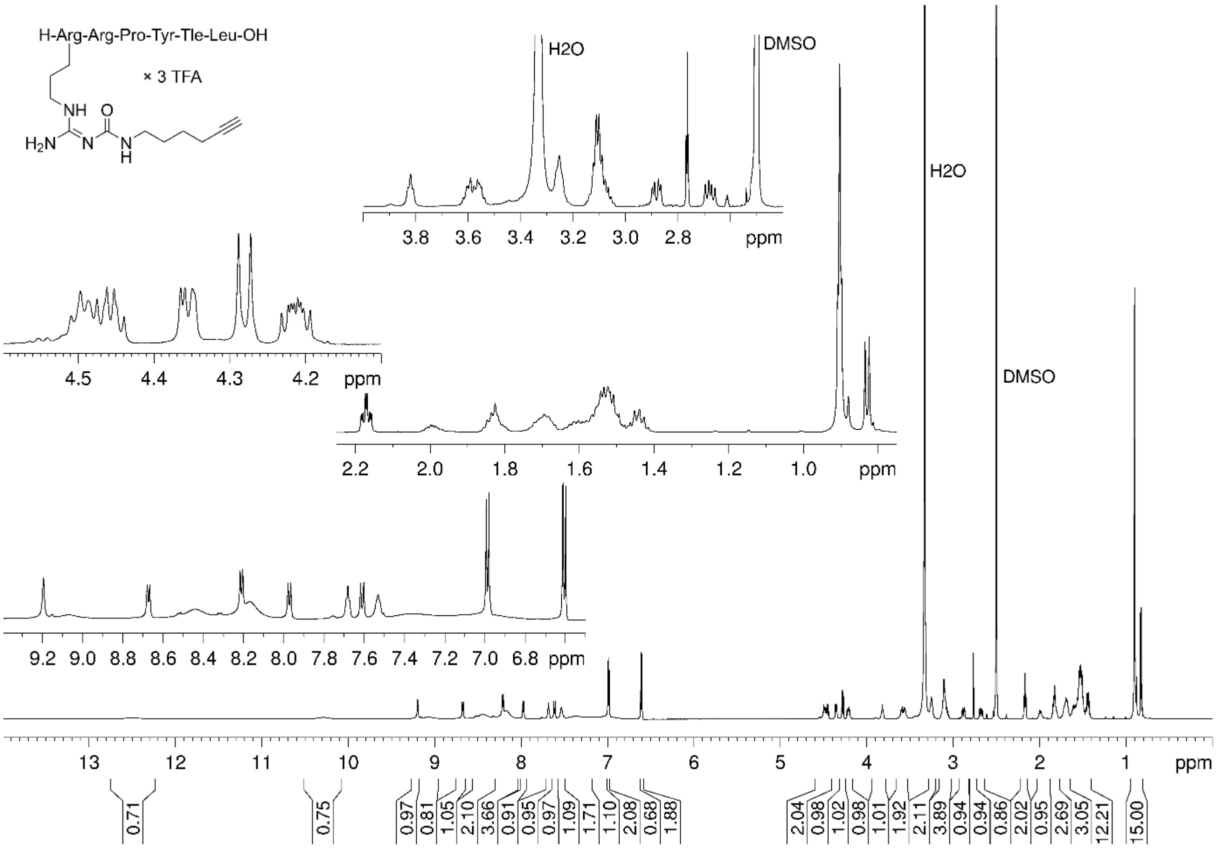

^{1}H-NMR spectrum (600 MHz, DMSO-d_6) of compound **7**

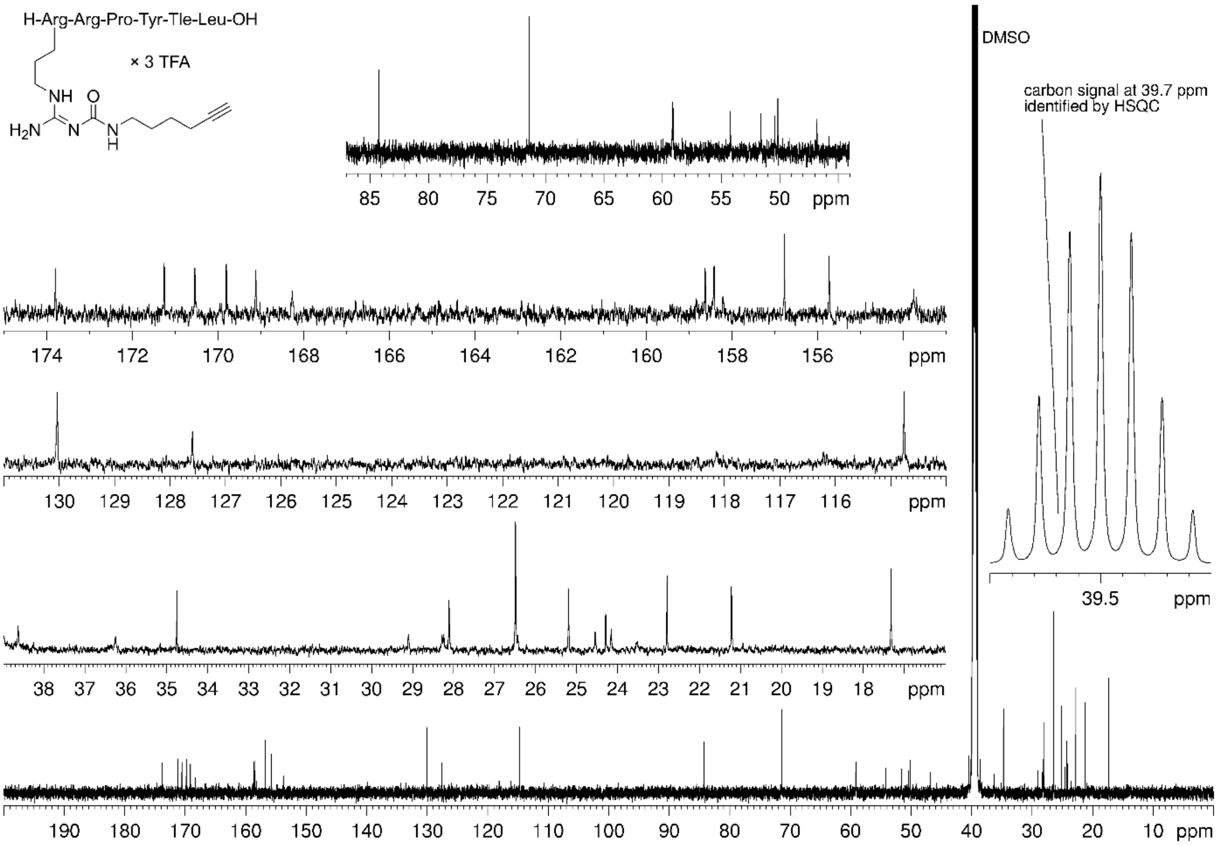

^{13}C-NMR spectrum (150 MHz, DMSO-d_6) of compound **7**

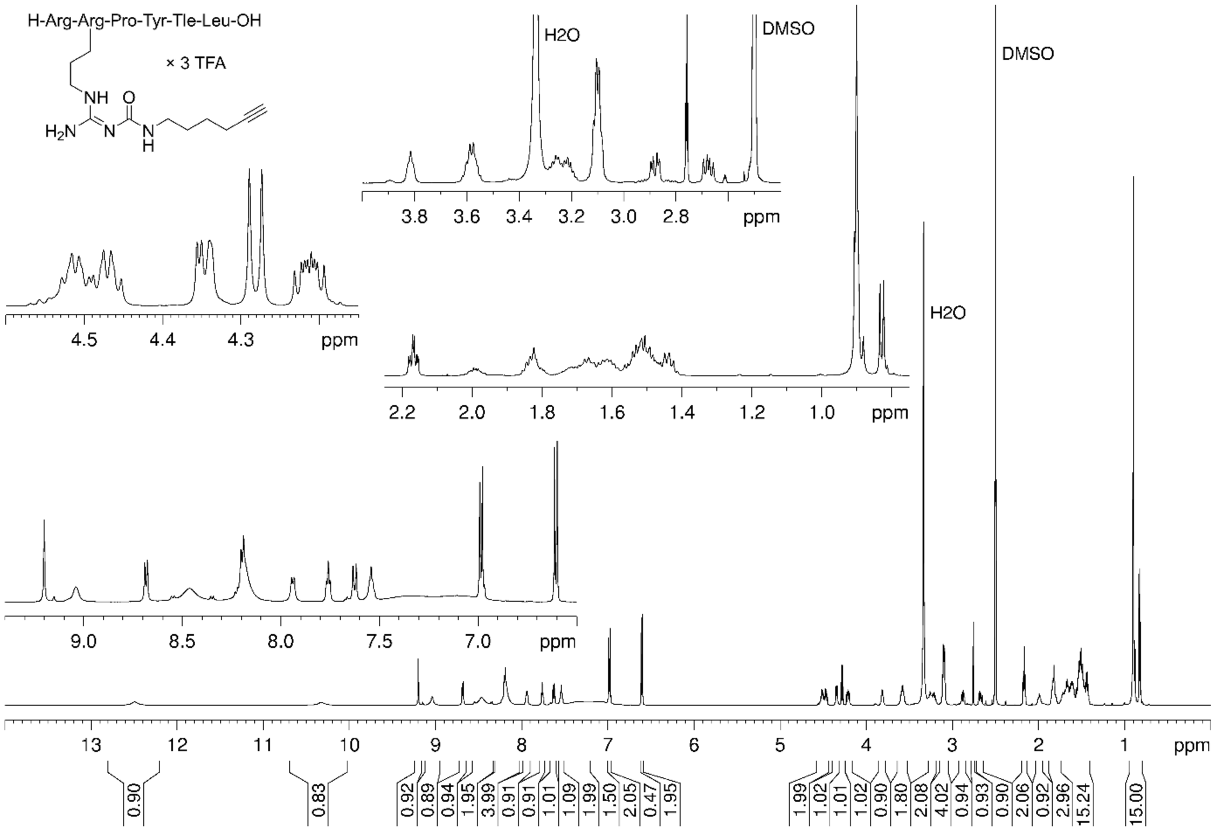

^{1}H-NMR spectrum (600 MHz, DMSO-d_6) of compound **8**

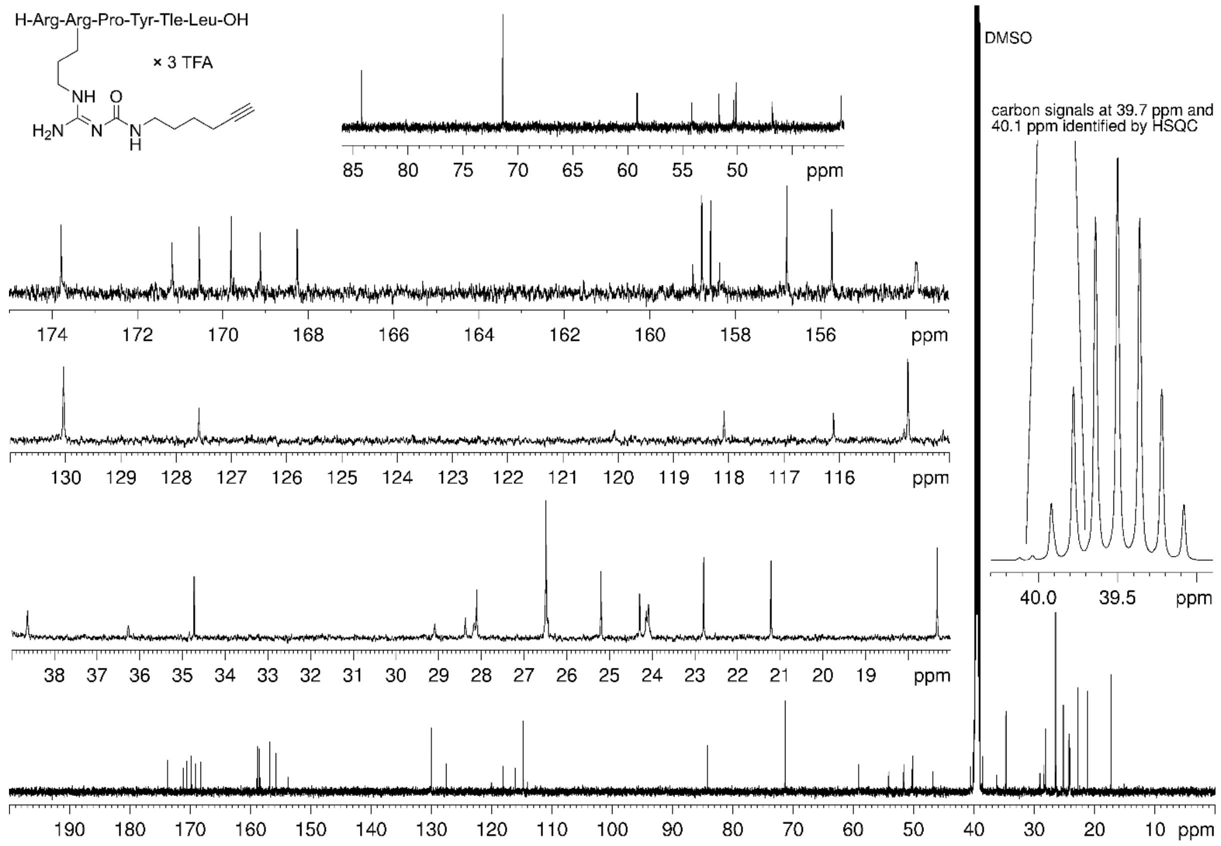

^{13}C-NMR spectrum (150 MHz, DMSO-d_6) of compound **8**

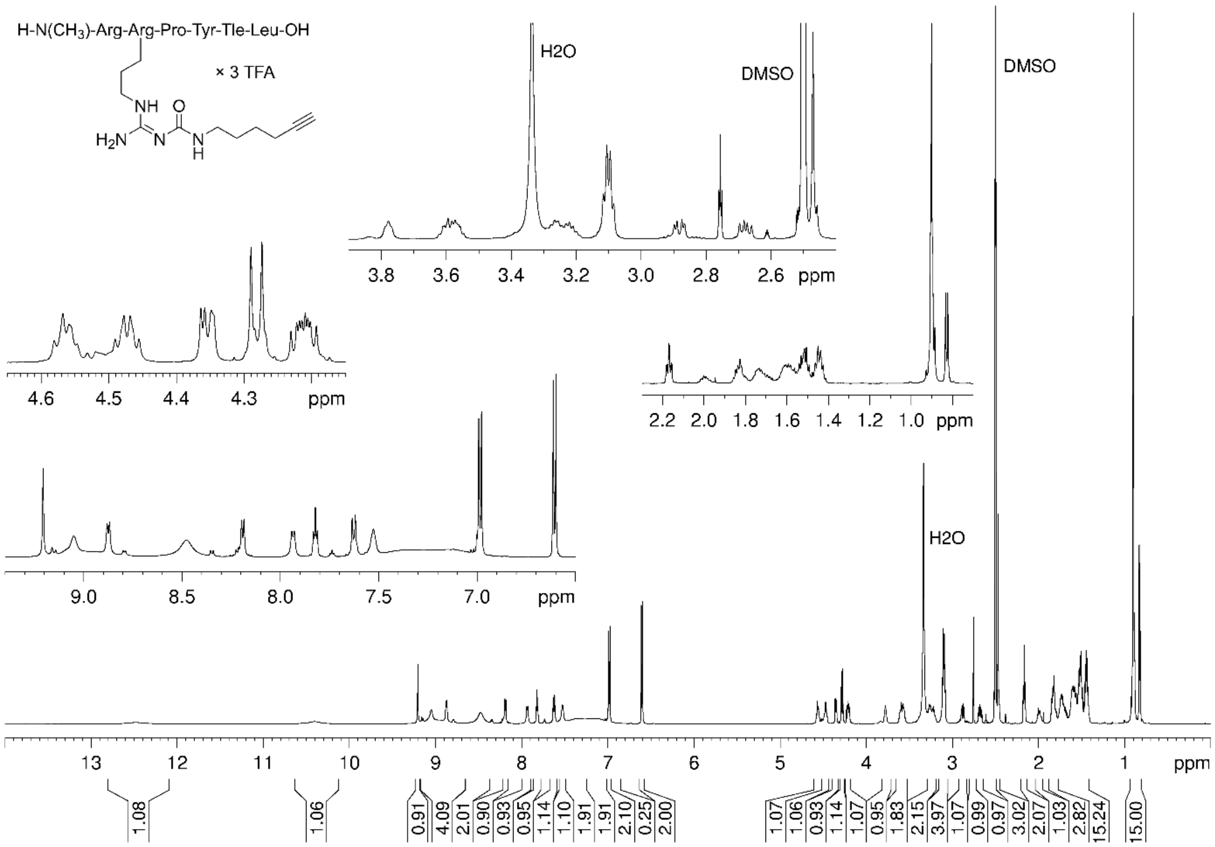

^{1}H-NMR spectrum (600 MHz, DMSO-d_6) of compound **9**

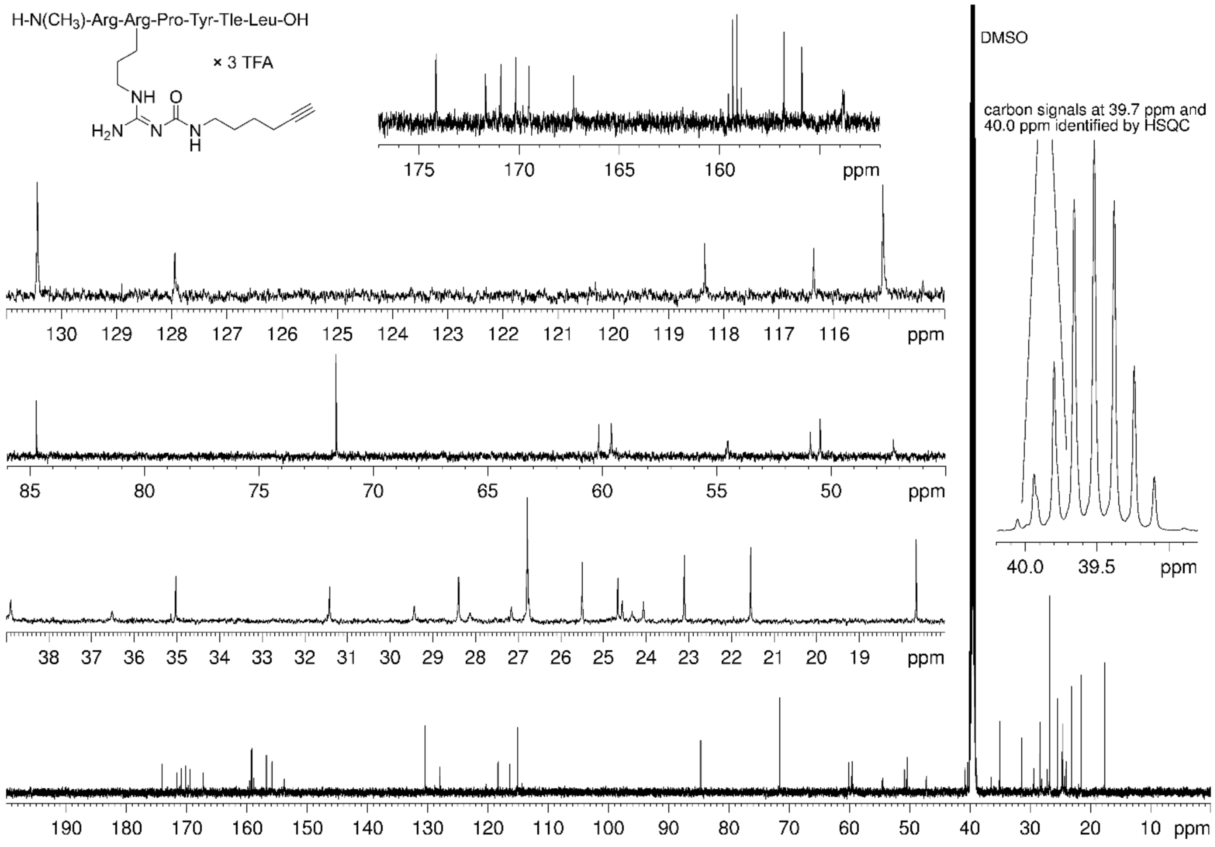

^{13}C-NMR spectrum (150 MHz, DMSO-d_6) of compound **9**

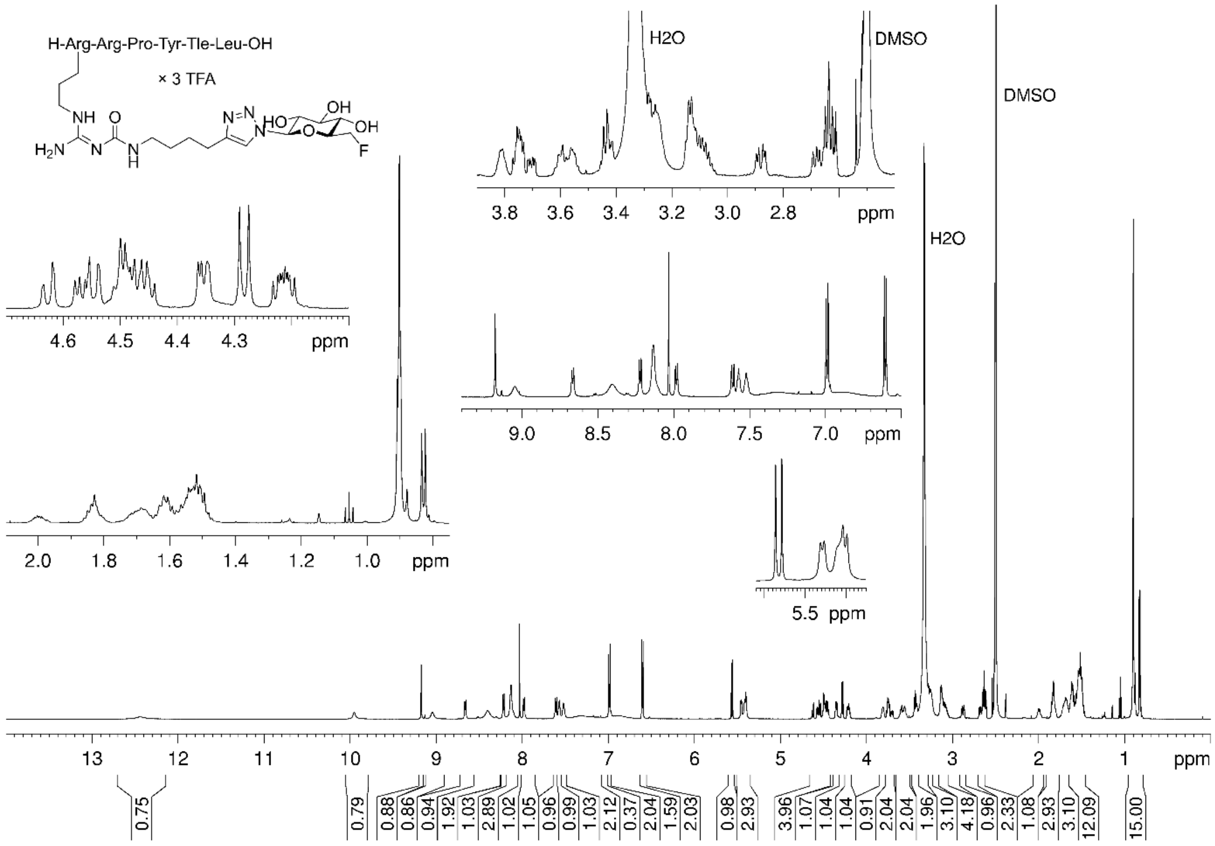

^{1}H-NMR spectrum (600 MHz, DMSO-d_6) of compound **11**

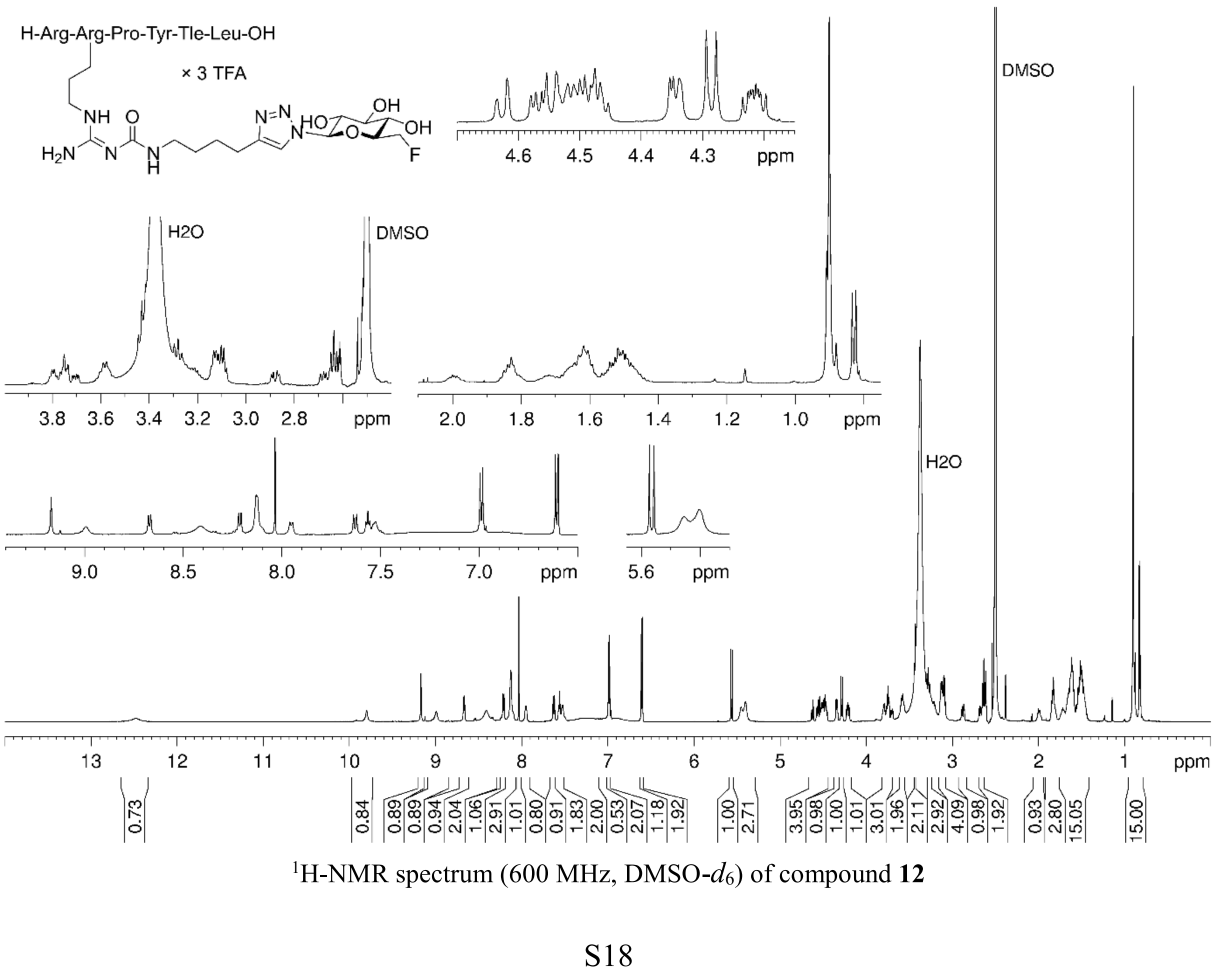

^{1}H-NMR spectrum (600 MHz, DMSO-d_6) of compound **12**

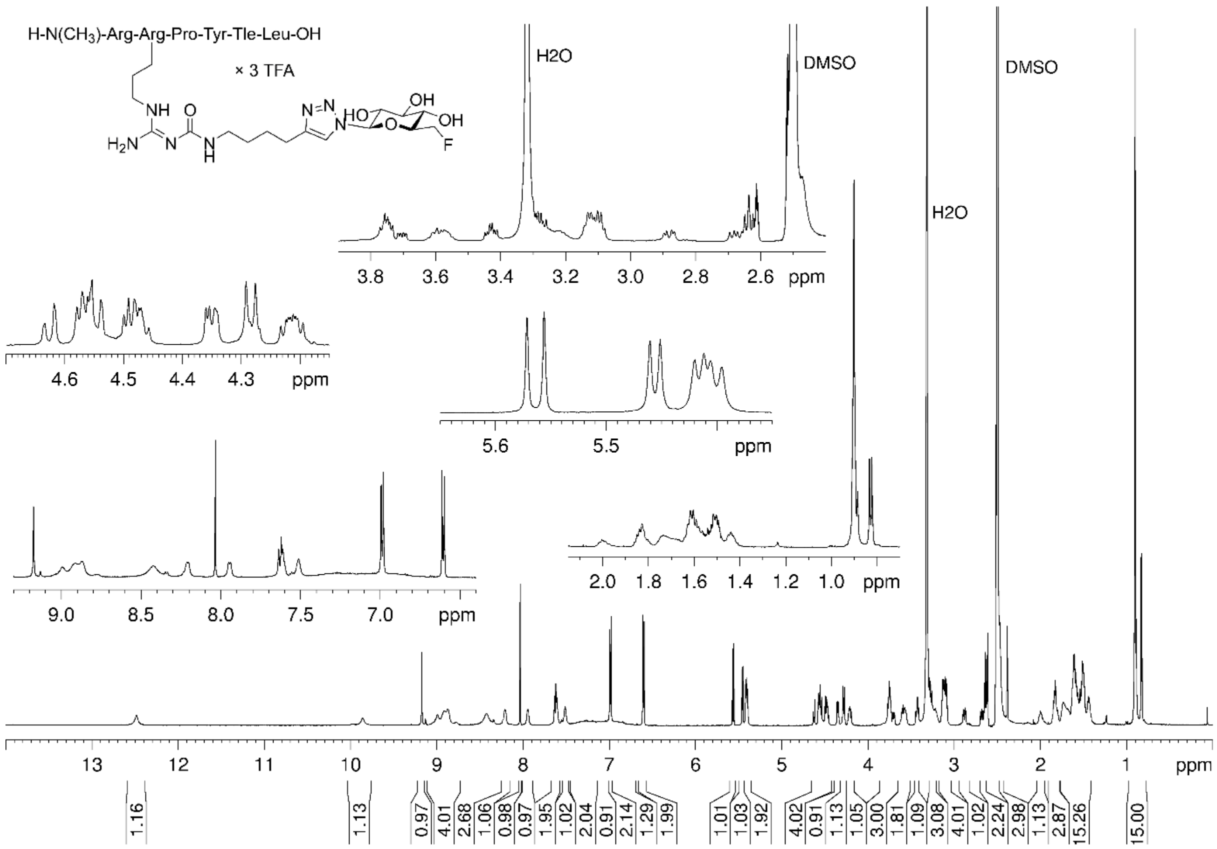

^{1}H-NMR spectrum (600 MHz, DMSO-d_6) of compound **13**

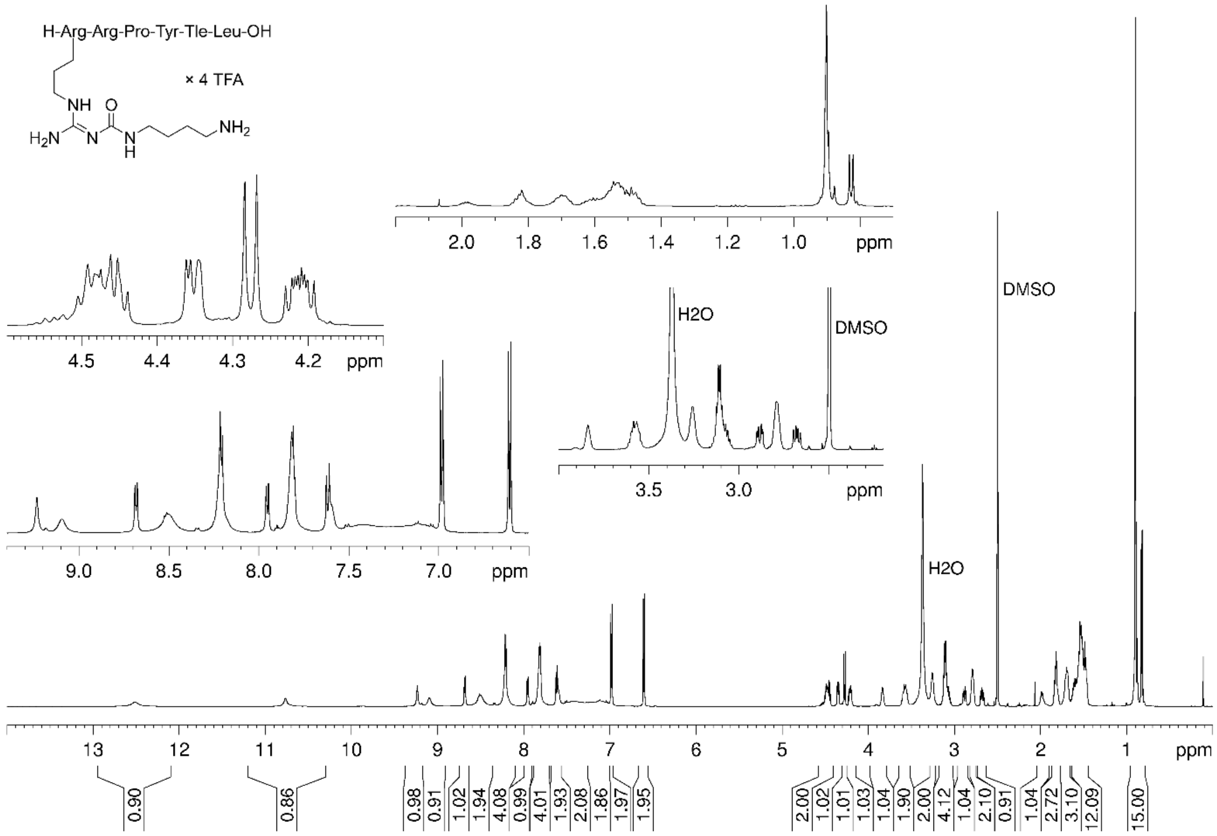

^{1}H-NMR spectrum (600 MHz, DMSO-d_6) of compound **14**

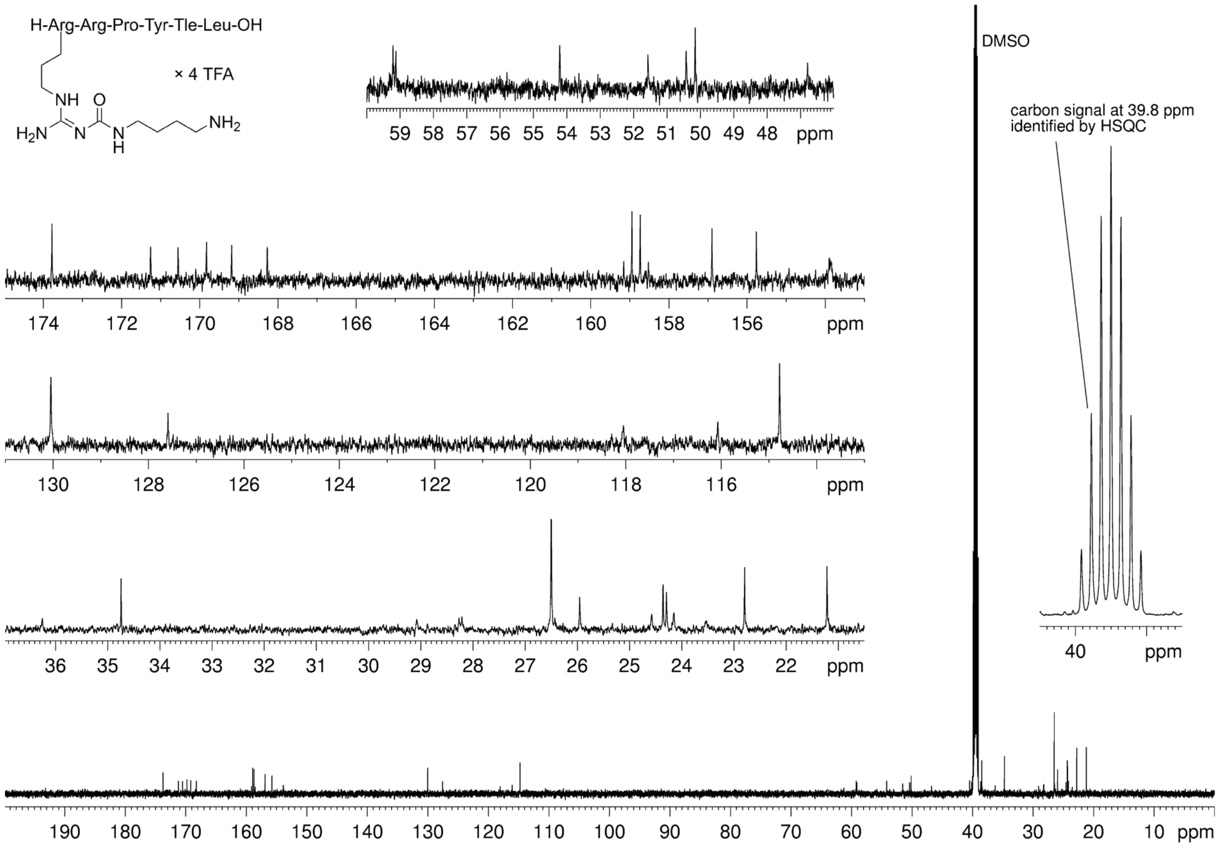

^{13}C-NMR spectrum (150 MHz, DMSO-d_6) of compound **14**

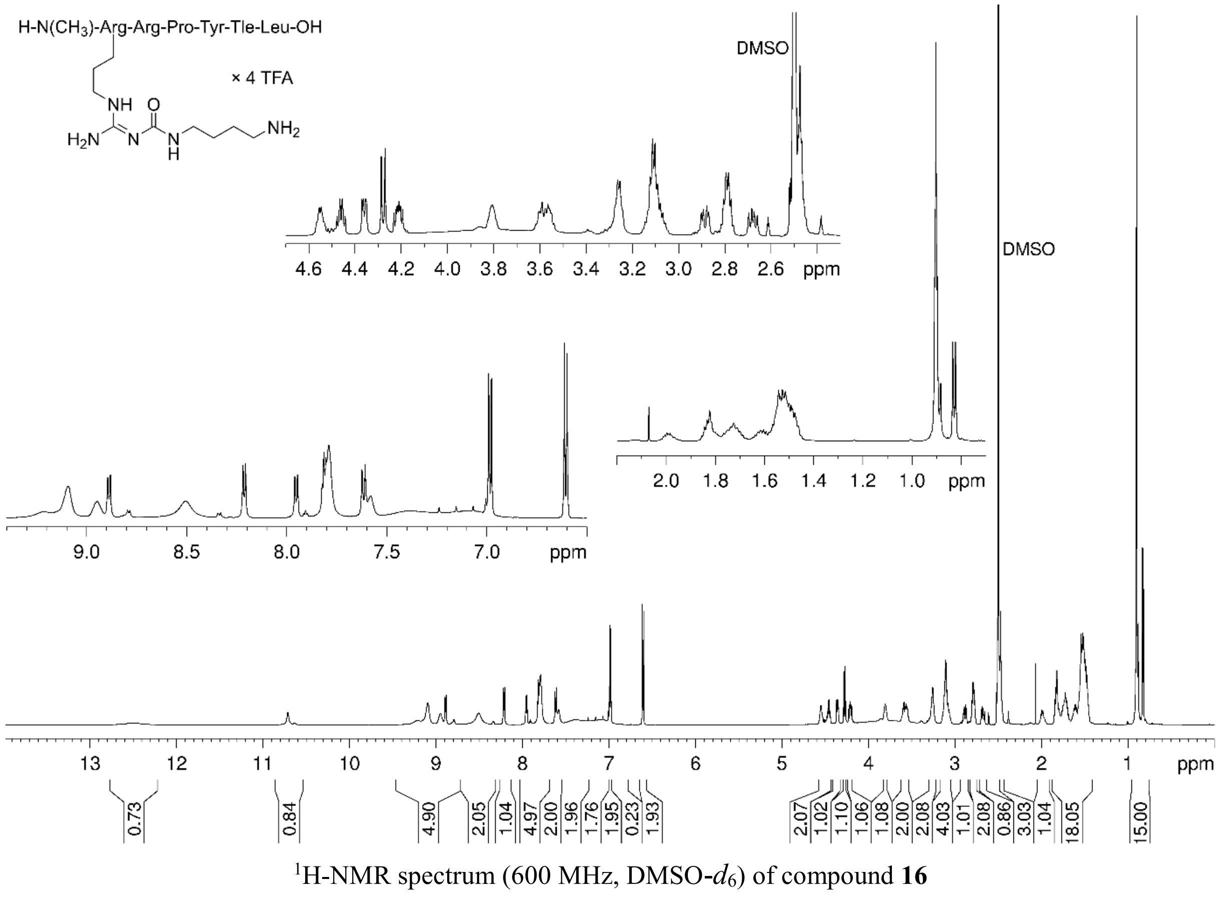

^{1}H-NMR spectrum (600 MHz, DMSO-d_6) of compound **16**

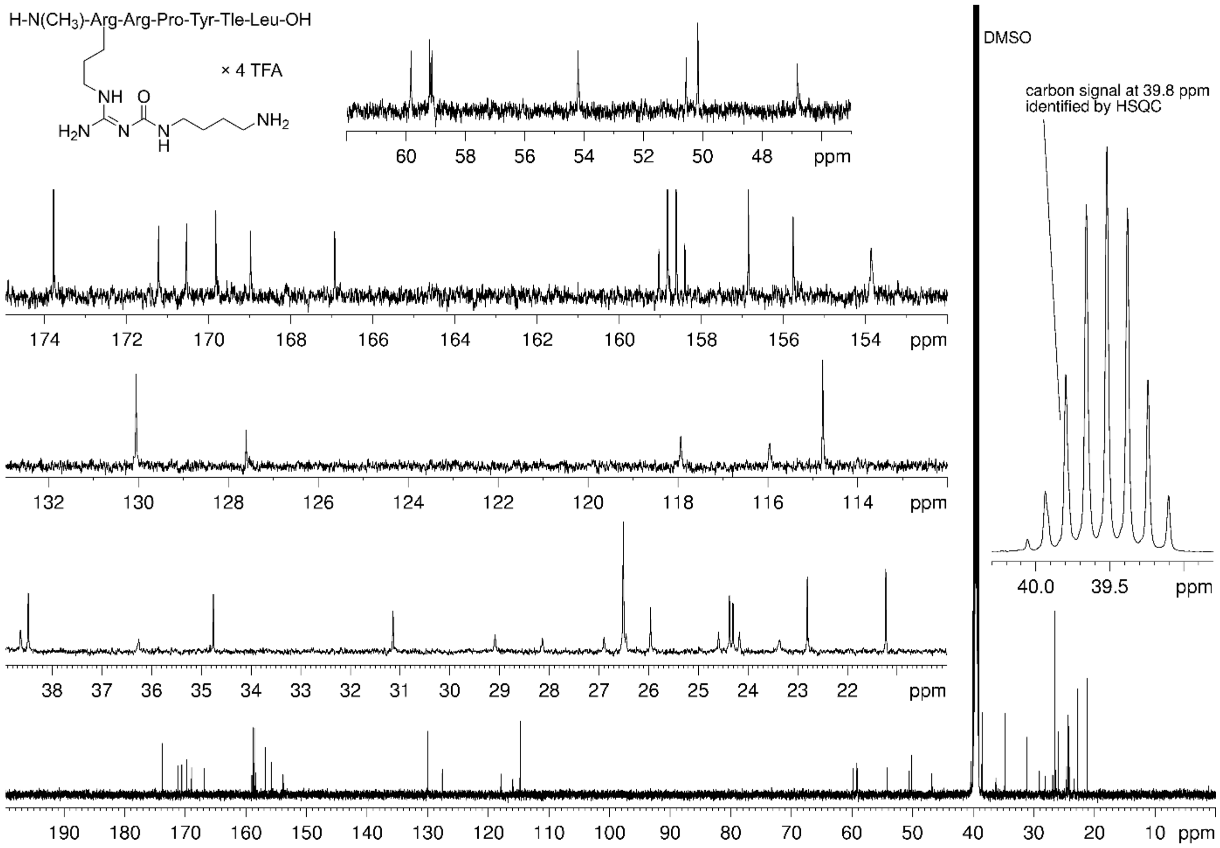

^{13}C-NMR spectrum (150 MHz, DMSO-d_6) of compound **16**

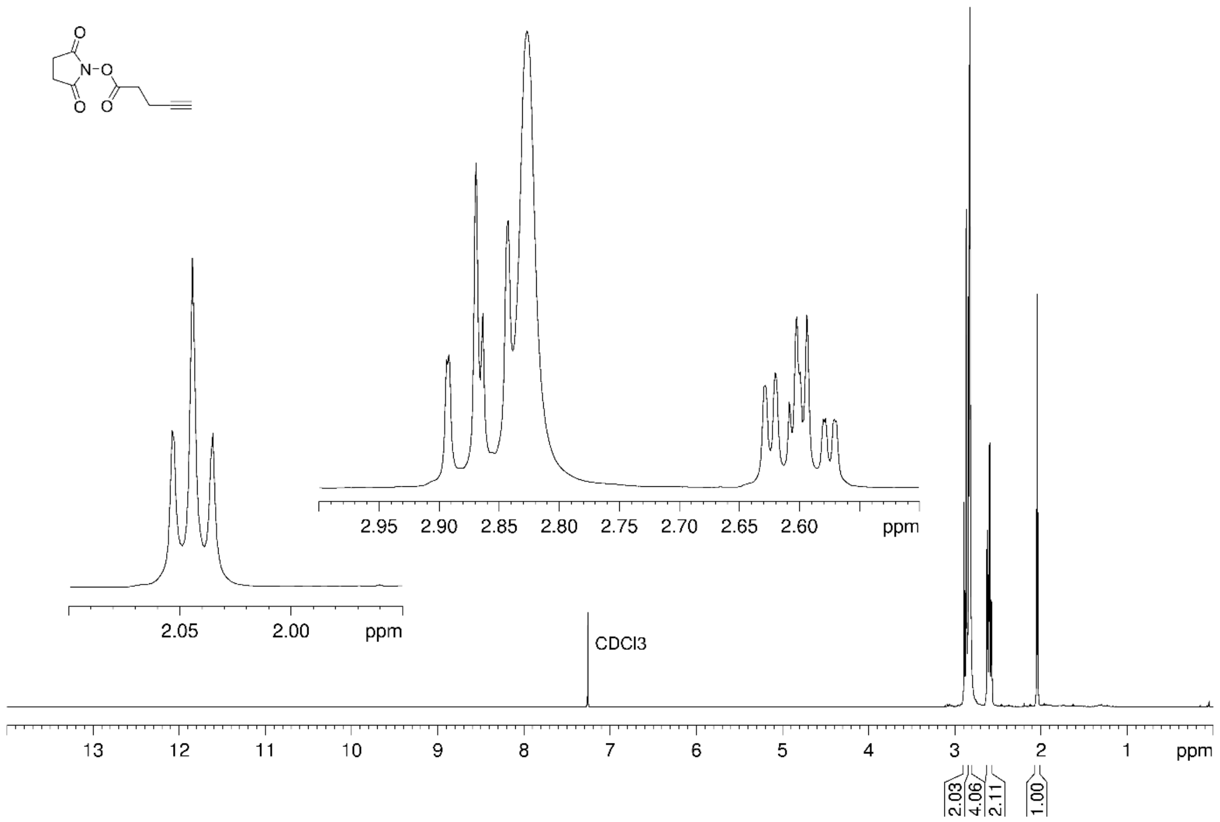

^{1}H-NMR spectrum (300 MHz, $CDCl_3$) of compound **17**

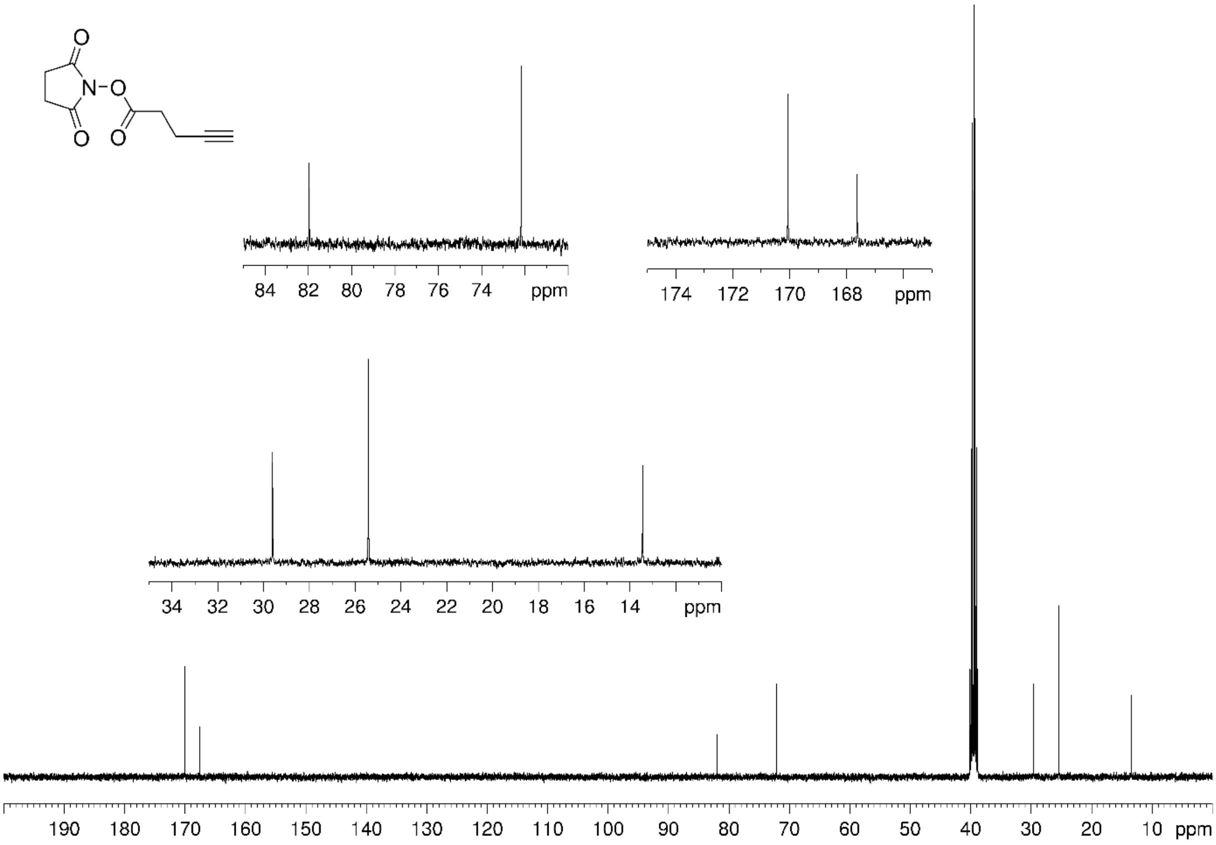

^{13}C-NMR spectrum (100 MHz, DMSO-d_6) of compound **17**

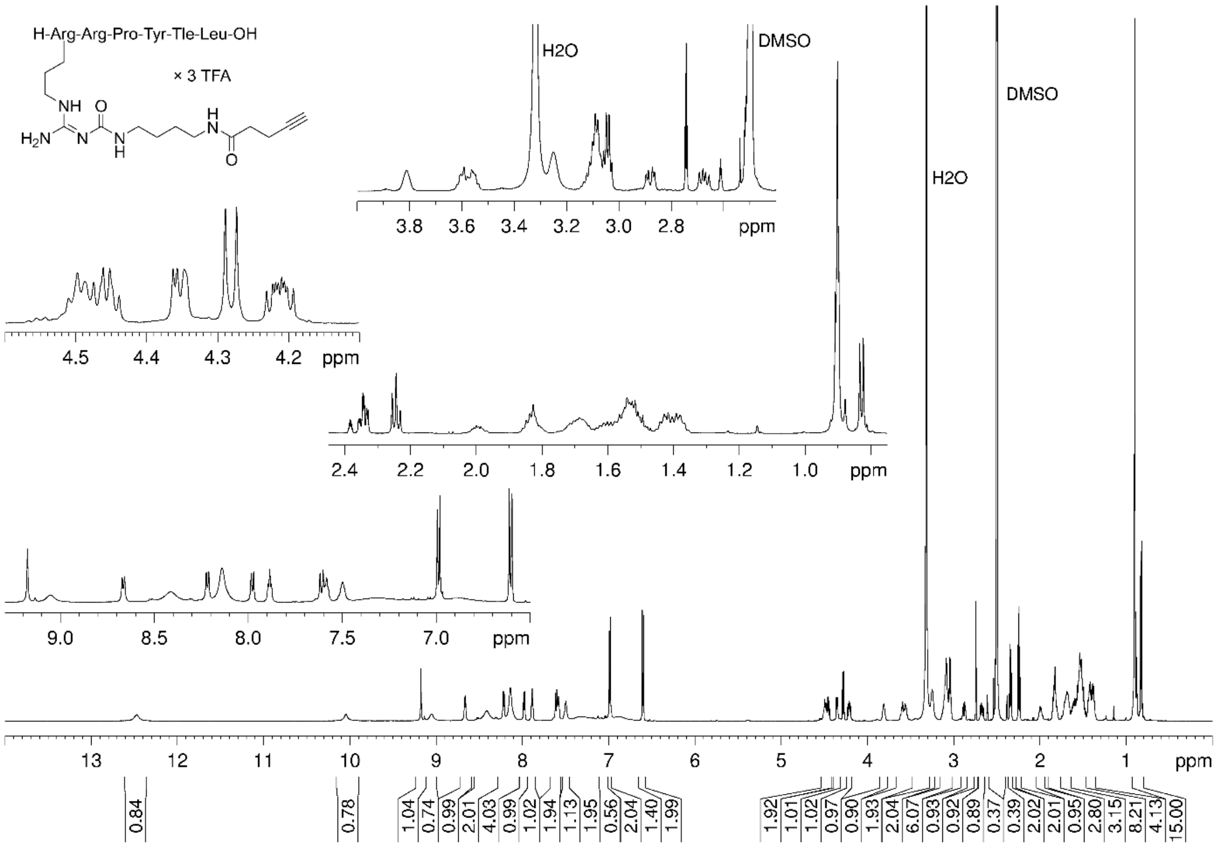

^{1}H-NMR spectrum (600 MHz, DMSO-d_6) of compound **18**

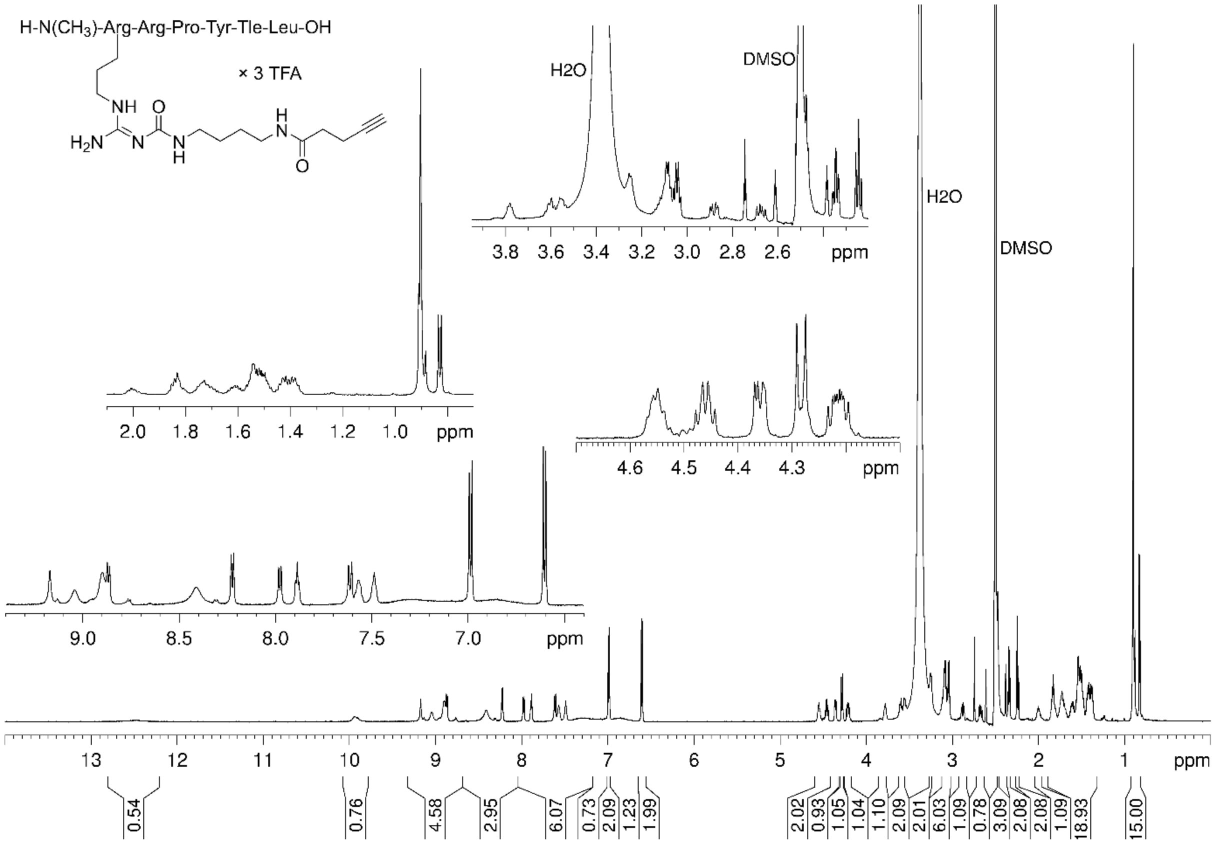

^{1}H-NMR spectrum (600 MHz, DMSO-d_6) of compound **19**

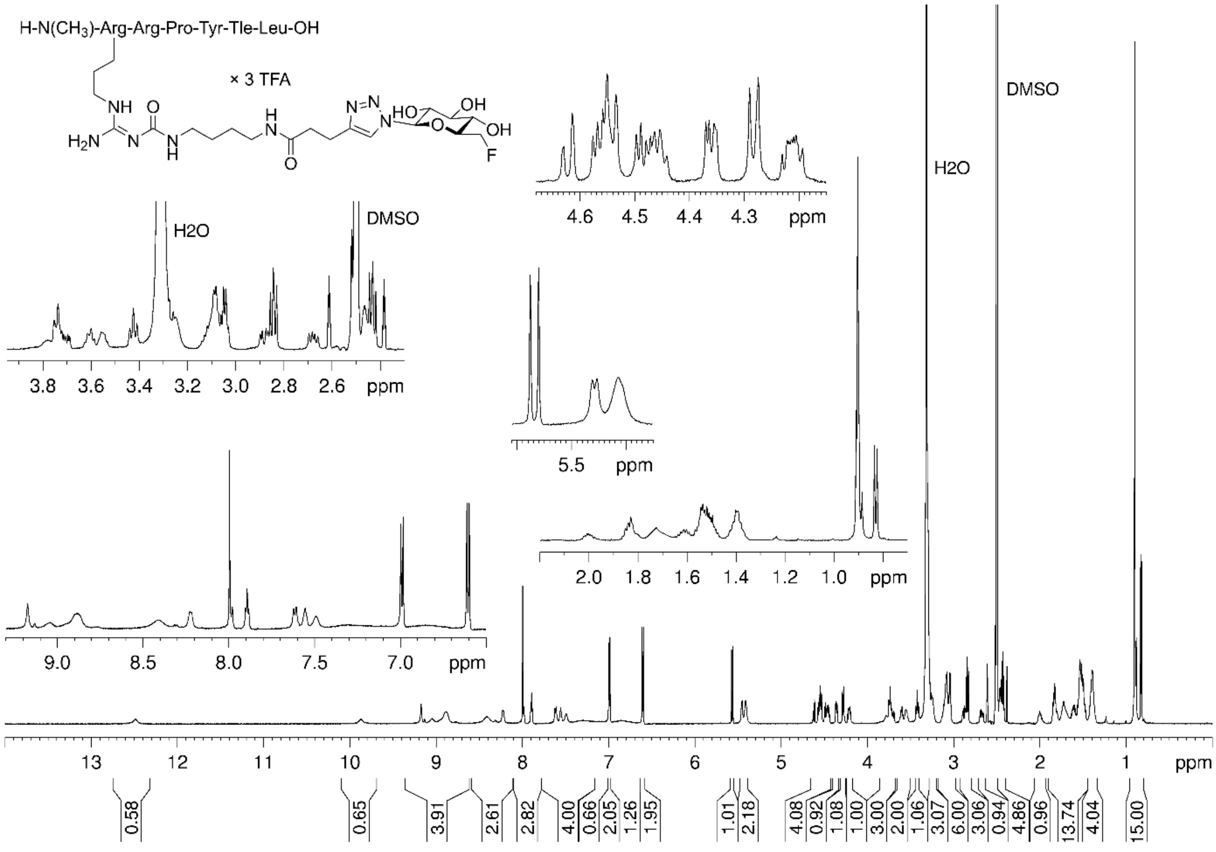

^{1}H-NMR spectrum (600 MHz, DMSO-d_6) of compound **21**

6. References

1. Keller, M.; Kuhn, K. K.; Einsiedel, J.; Hübner, H.; Biselli, S.; Mollereau, C.; Wifling, D.; Svobodova, J.; Bernhardt, G.; Cabrele, C.; Vanderheyden, P. M.; Gmeiner, P.; Buschauer, A. Mimicking of Arginine by Functionalized N(omega)-Carbamoylated Arginine As a New Broadly Applicable Approach to Labeled Bioactive Peptides: High Affinity Angiotensin, Neuropeptide Y, Neuropeptide FF, and Neurotensin Receptor Ligands As Examples. *J. Med. Chem.* **2016**, *59* (5), 1925-45.
2. Maschauer, S.; Einsiedel, J.; Hübner, H.; Gmeiner, P.; Prante, O. ^{18}F- and ^{68}Ga-Labeled Neurotensin Peptides for PET Imaging of Neurotensin Receptor 1. *J. Med. Chem.* **2016**, *59*, 6480-92.
3. Wang, M.; Zhang, H.; Wang, H.; Feng, H.; Deng, H.; Wu, Z.; Lu, H.; Li, Z. Development of [^{18}F]AlF-NOTA-NT as PET Agents of Neurotensin Receptor-1 Positive Pancreatic Cancer. *Mol. Pharm.* **2018**, *15*, 3093-3100.
4. Eaton, B.; Gold, L. Parallel SELEX allowing for asymmetrical reactions in combinatorial chemistry. **1999**, patent US5858660A.
5. Maschauer, S.; Haubner, R.; Kuwert, T.; Prante, O. ^{18}F-Glyco-RGD peptides for PET imaging of integrin expression: efficient radiosynthesis by click chemistry and modulation of biodistribution by glycosylation. *Mol. Pharm.* **2014,** *11*, 505-515.
